# Inequality in socio-emotional skills: A cross-cohort comparison^☆^

**DOI:** 10.1016/j.jpubeco.2020.104171

**Published:** 2020-11

**Authors:** Orazio Attanasio, Richard Blundell, Gabriella Conti, Giacomo Mason

**Affiliations:** Department of Economics, University College London, Gordon Street, London, WC1H0AX, UK; Institute for Fiscal Studies, UK

**Keywords:** Inequality, Socio-emotional skills, Cohort studies, Measurement invariance

## Abstract

We examine changes in inequality in socio-emotional skills very early in life in two British cohorts born 30 years apart. We construct comparable scales using two validated instruments for the measurement of child behaviour and identify two dimensions of socio-emotional skills: ‘internalising’ and ‘externalising’. Using recent methodological advances in factor analysis, we establish comparability in the inequality of these early skills across cohorts, but not in their average level. We document for the first time that inequality in socio-emotional skills has increased across cohorts, especially for boys and at the bottom of the distribution. We also formally decompose the sources of the increase in inequality and find that compositional changes explain half of the rise in inequality in externalising skills. On the other hand, the increase in inequality in internalising skills seems entirely driven by changes in returns to background characteristics. Lastly, we document that socio-emotional skills measured at an earlier age than in most of the existing literature are significant predictors of health and health behaviours. Our results show the importance of formally testing comparability of measurements to study skills differences across groups, and in general point to the role of inequalities in the early years for the accumulation of health and human capital across the life course.

## 1 Introduction

Human capital is a key determinant of economic growth and performance and of the resources an individual creates and controls over the life cycle (Hanushek and Woessmann, 2008). Human capital is also important for various determinants of individual well-being, ranging from life satisfaction to health (Conti et al., 2019). In recent years, the process of human capital accumulation has received considerable attention (Almond et al., 2018). There is growing consensus on the fact that human capital is a multidimensional object, with different domains playing different roles in labour market as well as in the determination of other outcomes, including the process of human development. It is also recognised that human capital is the output of a very persistent process, where early years inputs play an important and longlasting role (Cunha et al., 2010).

And yet, there are still large gaps in our knowledge of the process of human capital development. These gaps are partly driven by the scarcity of high quality longitudinal data measuring the evolution over the life cycle of different dimension of human capital. Moreover, there is a lack of consensus on the best measures and on the tools to collect high quality data. As a consequence, even when data are available in different contexts, their comparability is problematic (Richter et al., 2019).

In this paper, we focus on an important dimension of human capital, which has been receiving increasing attention in the last few years: socio-emotional skills. It has been shown that gaps in socio-emotional skills emerge at very young ages, and that in the absence of interventions are very persistent across the life cycle (Cunha et al., 2006). However, there is surprisingly little evidence on how inequality in this important dimension of human capital has changed across cohorts. In this paper, we start addressing this gap and focus on the measurement of these skills in two British cohorts: the one of children born in 1970 and the one of children born in 2000. We consider the measurement of socio-emotional skills during early childhood, as these skills have been shown, in a variety of contexts (Almlund et al., 2011) to have important long-run effects. Our goal is to characterise the distributions of socio-emotional skills in these cohorts and compare them. In the last part of the paper, we also consider the predictive power of different socio-emotional skills for health and socioeconomic outcomes.

We proceed in four steps. First, we construct a novel scale of childhood behavioural traits from two validated instruments and assess its comparability across cohorts. By performing exploratory and multiple-group factor analyses, we determine that two dimensions are a parsimonious representation of socio-emotional skills for both cohorts. Coherently with previous literature, we label them as ‘internalising’ and ‘externalising’ skills, the former relating to the ability of children to focus their drive and determination, and the latter relating to their ability to engage in interpersonal activities. Importantly, for the first time in economics, we study the comparability of the measures in the two cohorts. In particular, we test for *measurement invariance* of the items we use to estimate the latent factors. Intuitively, if one assumes that a set of measures is related to a latent unobserved factor of interest, one can think of this relationship as being driven by the saliency of each measure and the level. If one uses a given measure as the relevant metric for the relevant factor, its saliency will determine the scale of the factor, while some other parameters, which could be driven by the difficulty of a given test or the social norms and attitudes towards a certain type of behaviour, determine the *average level* of the factor. Comparability of estimated factors across different groups (such as different cohorts) assumes that both the parameters that determine the saliency of a given set of measures and those that determine the level of the factors do not vary across groups. We find that, for the measures we use and for both factors, we cannot reject measurement invariance for the saliency parameters. However, we strongly reject measurement invariance for the level parameters. These results imply that while we can compare the inequality in skills across the two cohorts, we cannot determine whether the *average levels* of the two factors are larger or smaller in one of them. While this result hinders a comparison in the level of skills, it is of interest per se to find that mothers of children born in England thirty years apart assess behaviours differently, so that differences in the raw scales cannot be unequivocally interpreted as differences in the underlying skills. We believe this is an important finding which deserves a greater degree of attention in the economic literature.

Second, given the results we obtain on measurement invariance, we proceed to compare the inequality in the two types of socio-emotional skills across the two cohorts, for both boys and girls. We find that the most recent cohort is more unequal in both dimensions of socio-emotional skills than the 1970 cohort. This result is particularly apparent for boys, and when looking at differences by maternal background. Third, we formally decompose the increase in inequality in skills into changes in the composition of maternal characteristics and changes in the returns to those characteristics, using recently developed methods based on Recentered Influence Function (RIF) regressions. In doing so, we provide the first application of this method to the child development literature.

Fourth, we study whether the socio-emotional skills we observe at a young age are an important determinant of a variety of adolescent (and adult, for the older BCS cohort) outcomes. We find that socio-emotional skills at age five are more predictive than cognitive skills for unhealthy behaviours like smoking and measures of health capital such as body mass index. The effect of cognition, instead, dominates for educational and labour market outcomes.

Our key contribution in this paper is to bring together two important strands of the literature: on the one hand, the literature on child development and early interventions; on the other hand, the literature on the measurement and the evolution of different types of inequality. While the former literature has provided robust evidence on the long-term impacts of a variety of early life circumstances, it has not systematically focused on describing and disentangling the sources of inequality in early human development; at the same time, the latter literature has carefully studied measures such as income, wages and wealth, overlooking other important - yet harder to measure - dimensions. In bridging these two literatures, we also apply recent methodological advances in factor analysis and show the importance of testing and constructing comparable aggregates. The methodology that we apply in this paper is likely to be relevant in many other settings, for example when measuring trends in inequality in other dimensions (such as satisfaction, mental health or well-being) whose measurement might have changed over time. Lastly, it is worth emphasizing that, while learning about the evolution and the determinants of inequality in socio-emotional skills is an interesting exercise in its own right, the ultimate goal of such research would be to uncover how much inequalities in early human development contribute to income or wealth inequality later in life. The present paper constitutes a first step towards such an endeavour.

The rest of the paper is organised as follows. We start in Section 2 by reviewing the main literature on determinants and consequences of socio-emotional traits. In Section 3, we briefly introduce the data we use in the analysis. In Section 4, we present the methods we use to identify the number of dimensions in socio-emotional skills and how we estimate the latent factors that represent them. In Section 5, we discuss the comparability of factors estimated with a given set of measures from different groups and the *measurement invariance* tests we use. Section 6 reports our empirical results on changes in inequality in socio-emotional skills and their predictive power for later outcomes. Section 7 concludes the paper.

## 2 Literature

The importance of cognition in predicting life course success is well established in the economics literature. However, in recent years the role played by ‘non-cognitive’ traits has been increasingly investigated. These traits include constructs as different as psychological and preference parameters such as social and emotional skills, locus of control and self-esteem, personality traits (e.g. conscientiousness), and risk aversion and time preferences. Given the vastness of this literature, we briefly review below the main papers on the determinants and consequences of socio-emotional traits which are more directly related to our work, and we refer to other sources for more exhaustive reviews (Borghans et al., 2008; Almlund et al., 2011; Goodman et al., 2015; Kautz et al., 2014).

### 2.1 Consequences of socio-emotional traits

One of the first papers to pioneer the importance of ‘non-cognitive’ variables for wages is Bowles et al. (2001). Heckman et al. (2006) suggest that non-cognitive skills are at least as important as cognitive abilities in determining a variety of adults outcomes. Lindqvist and Vestman (2011), using data based on personal interviews conducted by a psychologist during the Swedish military enlistment exam, show that both cognitive and noncognitive abilities are important in the labour market, but for different outcomes: low noncognitive abilities are more correlated with unemployment or low earnings, while cognitive ability is a stronger predictor of wages for skilled workers. Segal (2013), using data on young men from the US National Education Longitudinal Survey, shows that eight-grade misbehaviour is important for earnings over and above eight-grade test scores. Layard et al. (2014) find that childhood emotional health (operationalised using the same mother-reported Rutter scale we use in the 1970 British cohort study) at ages 5, 10 and 16 is the most important predictor of adult life satisfaction and life course success.

There are only few studies in economics specifically studying “non-cognitive” traits and health behaviours. Conti et al. (2010) and Conti et al. (2011) are the first to consider three early endowments, including child socio-emotional traits and health in addition to cognition, using rich data from the 1970 British cohort study. They find strong evidence that non-cognitive traits promote health and healthy behaviours, and than not accounting for them overestimates the effects of cognition; additionally, they document that child cognitive traits are more important predictors of employment and wages than socio-emotional traits or early health. Chiteji (2010) uses the US Panel Study of Income Dynamics (PSID) and finds that future orientation and self-efficacy (related to emotional stability) are associated with less alcohol consumption and more exercise. Cobb-Clark et al. (2014) use the Australian HILDA data and find that an internal locus of control (also related to emotional stability, perceived control over one's life) is related to better health behaviours (diet, exercise, alcohol consumption and smoking). Mendolia and Walker (2014) use the Longitudinal Study of Young People in England and find that individuals with external locus of control, low self-esteem, and low levels of work ethics, are more likely to engage in risky health behaviours. Prevoo and ter Weel (2015) construct measures of personality from maternal ratings at 10 and 16 in the British Cohort Study and find that their measure of conscientiousness is positively associated with education and economic outcomes, and negatively associated with body mass index and smoking. Goodman et al. (2015) review the interdisciplinary literature and provide a new analysis of the British Cohort Study, including a particular focus on the role of social and emotional skills (defined using a rich set of measurements of the age 10 sweep) in transmitting ‘top ‘job’ status between parents and their children. Savelyev and Tan (2019) show that the association between personality traits and health behaviours also holds in a high-IQ sample (the Terman Sample). Heckman et al. (2018) use, instead, early risky and reckless behaviours to measure socio-emotional endowments, and confirm their predictive power for education, log wages, smoking and health limits work.

Few papers attempt to make cross-cohorts comparisons about the importance of socio-emotional skills. Blanden et al. (2007) – one of the closest study to ours – examine cognitive skills, non-cognitive traits, educational attainment and labour market attachment as mediators of the decline in inter-generational income mobility in UK between the 1958 and the 1970 cohorts. The authors take great care in selecting non-cognitive items to be as comparable as possible across cohorts, from the Rutter scale at age 10 for the 1970 cohort and from the Bristol Social Adjustment Guide for the 1958 cohort; however, they do not carry out formal tests of measurement invariance and they do not construct factor scores fully comparable across cohorts as we do. Another paper related to ours is the one by Reardon and Portilla (2016), who study recent trends in income, racial, and ethnic school gaps in several dimensions of school readiness, including academic achievement, self-control, and externalising behavior, at kindergarten entry, using comparable data from the Early Childhood Longitudinal Studies (ECLS-K and ECLS-B) for cohorts born from the early 1990s to the 2000–2010 period in the US. They find that readiness gaps narrowed modestly from 1998 to 2010, particularly between high- and low-income students and between White and Hispanic students. Landersø and Heckman (2017) study the sources of differences in social mobility between US and Denmark; for the US, they use the antisocial, headstrong and hyperactivtity subscales from the Behavior Problem Index (BPI) in the Children of the NLSY79 (CNLSY), while for Denmark they use orderliness/organization/neatness grades from the Danish written exams.^1^ They find that, in both countries, cognitive and non-cognitive skills acquired by age 15 are more important for predicting educational attainment than parental income. Lastly, Deming (2017) uses two sets of skill measures and comparable covariates across survey waves for the NLSY79 and the NLSY97,^2^ and finds that the labour market return to social skills was much greater in the 2000s than in the mid-1980s and 1990s. Zilanawala et al. (2019) examine differences in socio-emotional and cognitive development among 11-year old children in the UK Millennium Cohort Study and the US Early Childhood Longitudinal Study-Kindergarten Cohort, and find that family resources explain some cross-national differences, however there appears to be a broader range of family background variables in the UK that influence child development. Importantly, none of these papers making comparisons across countries, cohorts or ethnic groups test for measurement invariance like we do.

### 2.2 Determinants of socio-emotional traits

Equally flourishing has been the literature on the determinants of child socio-emotional skills, which ranges from reduced-form, correlational or causal estimates, to more structural approaches. One of the first papers by (Segal, 2008) shows that a variety of family and school characteristics predict classroom behaviour. Carneiro et al. (2013) study the intergenerational impacts of maternal education, using data from the NLSY79 and an instrumental variable strategy; they find strong effects in terms of reduction in children's behavioural problems. Cunha et al. (2010) and Attanasio et al. (2020) both estimate production functions for child cognitive and socio-emotional development (in US and Colombia, respectively), and find an important role played by parental investments. Moroni et al. (2019) estimate production functions for child socio-emotional skills (internalising and externalising behaviour) at age 11 in the UK Millennium Cohort Study, and find that the effects of parental inputs which improve the home environment varies as a function both of the level of the inputs themselves and of the development of the child.

Interventions targeting Social and Emotional Learning (SEL) in a school setting have been shown to lead to significant improvements in socio-emotional skills, attitudes, behaviours, and academic performance (Durlak et al., 2011), and a substantial positive return on investments (Belfield et al., 2015); after-school programs have been proved to be equally effective (Durlak et al., 2010).

Additionally, it has been shown that a key mechanism through which early childhood interventions improve adult socioeconomic and health outcomes is by boosting socio-emotional skills, measured as four teacher-reported behavioural outcomes in the project STAR^3^ (Chetty et al., 2011), reductions in externalising behaviour (from the Pupil Behavior Inventory) at ages 7–9 in the Perry Preschool Project (Heckman et al., 2013; Conti et al., 2016), or improvements in task orientation at ages 1–2 in the Abecedarian Project (Conti et al., 2016).

In sum, even if the literature on the determinants and consequences of socio-emotional skills has been booming, most papers use skills measured in late childhood or in adolescence; and no paper in economics formally tests for invariance of measurements across different groups and constructs fully comparable scores. In this paper, we use measures of child socio-emotional development at age 5, hence before the start of elementary school; and we construct comparable scales across the two cohorts we study (the 1970 and the 2000 British cohorts), so that we can investigate changes in inequality in early development, their determinants, and consequences, in a parallel fashion.

## 3 Data

We use information from two nationally representative longitudinal studies in the UK, which follow the lives of children born approximately 30 years apart: the British Cohort Study (BCS) and the Millennium Cohort Study (MCS). The BCS includes all individuals born in Great Britain in a single week in 1970. The cohort members' families – and subsequently the members themselves – were surveyed on multiple occasions. For this paper we augment the information collected at the five-year survey with data from birth, adolescence (16), and adulthood (30, 38, 42). The MCS follows individuals born in the UK between September 2000 and January 2002. We use the first survey – carried out at 9 months of age – and the sweeps at around 5 and 14 years of age.^4^

Our main focus is on socio-emotional skills of children around age five. We take advantage of the longitudinal nature of the cohorts by merging information from surveys before and after age five. From the birth survey, we include information on gestational age and weight at birth, previous stillbirths, parity, maternal smoking in pregnancy, maternal age, height, and marital status. From the five year survey, we extract maternal education, employment status, and the father's occupation. All the above variables are transformed or recoded to maximise comparability between the two studies. Furthermore, we add some adolescent outcomes such as smoking and BMI, with the caveat that these are surveyed at different ages – 16 in BCS and 14 in MCS. Finally, for the 1970 cohort we also include measures of adult educational attainment, BMI, and income. Variable definitions are available in Table A1.

Ideally, we would compare socio-emotional skills alongside cognitive skills. However, the cognitive tests administered to each cohort have no overlap, even at the item level. We thus use the available cognitive tests in each cohort to estimate simple confirmatory factor models with a single latent dimension, separately by cohort (see Table A1 for the tests used). Unlike the other indicators in our analysis, cognitive skills are thus not comparable across cohorts.

Another complication arises from the fact that, differently from the British Cohort Study, the Millennium Cohort Study has a stratified design. It oversamples children living in administrative areas characterised by higher socioeconomic deprivation and larger ethnic minority population (Plewis et al., 2007). We rebalance the MCS sample to make it nationally representative by excluding from the analysis a fraction of observations from the oversampled areas, proportionally to their sampling probability.^5^ Finally, we also restrict our sample to individuals born in England and to cases where there is complete information on socio-emotional skills at five years of age. The final sample contains 9545 individuals from the British Cohort Study, and 5572 from the Millennium Cohort Study. Summary statistics for the full and estimation samples are displayed in Table 1. After the rebalancing step, the MCS estimation sample closely mirrors the full sample in terms of average observable characteristics, thus preserving representativeness.

**Table 1:** Summary statistics.

	Estimation sample		Full sample	
	BCS	MCS	BCS	MCS
	*N*=9545	*N*=5572	*N*=14063	*N*=11530
	Mean (SD)	Mean (SD)	Mean (SD)	Wt. Mean (SD)
Mother age	25.92 (5.35)	29.43 (5.67)	25.93 (5.48)	28.96 (5.91)
Mother height (m)	1.61 (0.06)	1.64 (0.07)	1.61 (0.06)	1.64 (0.07)
Unmarried	0.05	0.36	0.07	0.38
Nonwhite child	0.03	0.11	0.04	0.14
Firstborn child	0.38	0.42	0.37	0.42
Number previous stillbirths	0.02 (0.15)	0.01 (0.10)	0.02 (0.16)	0.01 (0.11)
Mother smoked in pregnancy	0.39	0.20	0.40	0.22
Preterm birth	0.04	0.07	0.05	0.07
Missing gest. age	0.19	0.01	0.20	0.01
Birthweight (kg)	3.31 (0.53)	3.38 (0.58)	3.27 (0.58)	3.36 (0.59)
Five-year survey				
Number of children in the household	1.55 (1.13)	1.34 (0.99)	1.56 (1.14)	1.35 (1.10)
Mother has post-compulsory education	0.38	0.57	0.38	0.45
Mother is employed	0.42	0.62	0.42	0.60
Father occupation: blue collar	0.61	0.41	0.62	0.41
No father figure	0.05	0.17	0.05	0.18

*Notes*: The table shows the mean values of harmonised variables (and the standard deviation, for continuous ones). The *estimation sample* is the subsample used in the analysis. The *full sample* is the entire sample of children in both cohorts, residing in England at birth. Mean estimates for the full sample in the MCS cohort are weighted to account for survey design.

## 4 Dimensions of socio-emotional skills

Child socio-emotional skills are an unobservable and difficult to measure construct. Over recent years, the measurement of such skills has evolved and, over time, different measures have been used. As we discuss below, this makes the comparison of socio-emotional skills across different groups, assessed with different tools, difficult.

A common approach to infer a child's socio-emotional development is based on behavioural screening scales. As part of these tools, mothers (or teachers) indicate whether their children exhibit a series of behaviours – the *items* of the scale. In the British and Millennium Cohort Studies, two different scales were employed. In the BCS, the Rutter A Scale was used (Rutter et al., 1970) while in the MCS mothers were administered the Strengths and Difficulties Questionnaire (SDQ, Goodman, 1994, Goodman, 1997). The SDQ was created as an update to the Rutter scale. It encompasses more recent advances in child psychopathology, and emphasises positive traits alongside undesirable ones (Stone et al., 2010). Goodman (1997) administered both scales to a sample of children, and showed that the scores are highly correlated, and the two measures do not differ in their discriminatory ability. The Rutter and SDQ scales are reproduced in Table A2; they have 23 and 25 items each, respectively. In the child psychiatry and psychology literatures, the Rutter and SDQ scales are regarded as measures of behavioural problems and mental health. However, in our analysis we follow the economics literature, and - after having recoded them accordingly - we interpret them as measures of positive child development (Goodman and Goodman, 2011).

While the Rutter and SDQ scales are similar in their components (since the latter was developed from the former, see Goodman (1994)), there is no a priori reason to expect them to be directly comparable. First, the overlap of behaviours described in the two scales is only partial, given that - by design - the SDQ includes also strengths, in addition to weaknesses. Second, the wording of each item is slightly different, both in the description and in the options that can be selected as answers. Third, the different ordering of the items within each scale might lead to order effects. Fourth, and no less importantly, the interpretation of each behaviour by respondents living 30 years apart (1975 vs 2006) might differ due to a host of evolving societal norms. Nonetheless, the level of comparibility of the two scales is higher than that of other scales used in comparative work in the literature reviewed in Section 2.

As our goal is to compare socio-emotional skills across the two cohorts, we construct a new scale by retaining the items that are worded in a similar way across the two original Rutter and SDQ scales, and making some slight coding adjustments to maximise comparability. In what follows, we will consider the included items to be the same *measure* in the two cohorts. The wording of the items we will be using in the analysis is presented in Table 2: we retain 13 items for the BCS (two of them are grouped) and 11 for the MCS with high degree of comparability. We exclude from the analysis items that were completely different between the two questionnaires to maximise comparability between the two cohorts, as it is standard good practice in the psychometric literature (see for example Kern et al. (2014)).^6^ More details on the derivation of the scale are available in Appendix A.

**Table 2:** Subscale of comparable items.

Itm.	Factor	Cat.	Title	Rutter wording (BCS 1970)	SDQ wording (MCS 2000/1)
1	EXT	3	*Restless*	Very restless. Often running about or jumping up and down. Hardly ever still	Restless, overactive, cannot stay still for long
2	EXT	3	*Squirmy/fidgety*	Is squirmy or fidgety	Constantly fidgeting or squirming
3	EXT	3	*Fights/bullies*	Frequently fights other children + Bullies other children	Often fights with other children or bullies them
4	EXT	3	*Distracted*	Cannot settle to anything for more than a few moments	Easily distracted, concentration wanders
5	EXT	2	*Tantrums*	Has temper tantrums	Often has temper tantrums or hot tempers
6	EXT	2	*Disobedient*	Is often disobedient	(+) Generally obedient, usually does what adults request
7	INT	3	*Worried*	Often worried, worries about many things	Many worries, often seems worried
8	INT	3	*Fearful*	Tends to be fearful or afraid of new things or new situations	Nervous or clingy in new situations, easily loses confidence
9	INT	3	*Solitary*	Tends to do things on his/her own, rather solitary	Rather solitary, tends to play alone
10	INT	3	*Unhappy*	Often appears miserable, unhappy, tearful or distressed	Often unhappy, down-hearted or tearful
11	INT	2	*Aches*	Complains of headaches + Complains of stomach-ache or has vomited	Often complains of head- aches, stomach-ache or sickness

*Notes*: *Itm.* is item number. *Factor* is the latent construct to which the item loads – EXT is Externalising skills, INT is Internalising skills. *Cat.* is the number of categories in which the item is coded – 2 denotes a binary item (applies/does not apply) and 3 denotes a 3-category item. *Title* is a short label for the item. *Wording* columns show the actual wording in the scales used in each of the cohort studies. Items denoted by (+) are positively worded in the original scale.

Item-level prevalence by cohort and gender is in Table A3. We see that, in general, there are more similarities across genders within the same cohort, than across cohorts. For the majority of items, there is a lower prevalence of problematic behaviours in the MCS than in the BCS; however, four items (distracted, tantrums, fearful, aches) show a higher prevalence in 2006 than in 1975. Regardless, a simple cross-cohort comparison of item-level prevalence is misleading because of changing perceptions and norms about what constitutes problematic behaviour in children. The analysis in Section 5 tackles this issue.

In the remainder of this section, we analyse the properties of the new scale. Following a common approach, we proceed in two steps. First, we carry out an *exploratory* step, where we study the factor structure of our scale. The aim of this step is to examine the correlation between observed measures in a data-driven way, imposing the least possible assumptions. Here, we establish how many latent dimensions of socio-emotional skills the scale is capturing, and which items of our scale are measuring which dimension. As a second step, we set up a *confirmatory* factor model. This model fixes the number of latent dimensions, and imposes a dedicated measurement structure, based on the insights obtained in the exploratory step. This is the model to which we apply the measurement invariance analysis of Section 5.

### 4.1 Exploratory analysis

The original Rutter scale, used in the BCS cohort, distinguishes behaviours into two subscales: *anti-social* and *neurotic* (Rutter et al., 1970). This two-factor conceptualisation has been validated using data from multiple contexts, and the latent dimensions have been broadly identified as externalising and internalising behaviour problems.^7^ The Strength and Difficulties Questionnaire, used in the MCS cohort, was instead conceived to have five subscales of five items each. The five subscales are: *hyperactivity*, *emotional symptoms*, *conduct problems*, *peer problems*, and *prosocial*. This five-factor structure has been validated in many contexts (Stone et al., 2010); lower-dimensional structures have been also suggested (Dickey and Blumberg, 2004). Recent research has shown that there are some benefits to using broader subscales that correspond to the externalising and internalising factors in Rutter, especially in low-risk or general population samples (Goodman et al., 2010). Indeed, the internalising and externalising dimensions were introduced in psychology by Achenbach (1966), who showed that they are the two main factors underlying a wide range of psychological measures; as noted in Achenbach et al. (2016), more than 75,000 articles have been published on internalising and externalising problems.

We use exploratory factor analysis (EFA) to assess the factor structure of our new scale, composed of 11 items of the Rutter scale in the BCS and the corresponding items of the SDQ in the MCS.^8^ We start by investigating the number of latent constructs that are captured by the scale, using different methods developed in the psychometric literature, and recently adopted by the economics literature. The results are displayed in Table A4. As pointed out in Conti et al. (2014), there is relatively little agreement among procedures; this is the case especially for the Rutter items in the BCS data, where different methods suggest to retain between 1 and 3 factors, while most methods suggest to retain 2 factors for the SDQ items in the MCS.

Given the test results, we perform a series of exploratory factor analyses, assuming a one-, two- or three-factor structure, respectively. The results for the 1-factor solution, reported in Table A5, show relatively similar loadings for both males and females across the two cohorts, of slightly bigger magnitude for the last four items in the MCS than in the BCS; thus, we retain the 1-factor solution for the measurement invariance analysis, in the first instance. The results for the 3-factor solution, instead, also reported in Table A5, show a less homogeneous picture^9^: while the magnitude of the loadings is relatively similar across the two cohorts for the first factor, items 3 and 5 only load on the second factor for the MCS, not for the BCS; more importantly, the EFA clearly shows that the third factor only loads on one single item (item number 9, “solitary”) for both cohorts. Given that a one-item factor implies that the item perfectly proxies for the factor, we are not able to test for measurement invariance in this case. Hence, the 3-factor solution is not supported by our EFA results. Last, the two-factor EFA is shown in Table A6 and delivers a neat and sensible separation between items: similarly-worded items load on the same factor across the two cohorts, and also the magnitude of the respective loadings (measuring the strength of the association between the item and the factor) is very similar. Following previous research, we name the first dimension *Externalising skills* (EXT, indicating low scores on the items restless, squirmy/fidgety, fights/bullies, distracted, tantrums, and disobedient) and the second dimension *Internalising skills* (INT, indicating low scores on the items worried, fearful, solitary, unhappy, and aches).^10^

### 4.2 Factor model

After having studied the factor structure underlying the 11 common items in the previous section, we now specify a multiple-group factor analysis model to formally quantify the strength of the relationship between the observed items in our scale and the latent socio-emotional skills, and to test for invariance across cohorts. We specify two groups of children *c* = {*BCS*, *MCS*}, corresponding to the two cohorts. Each individual child is denoted by *j*=1…*N*_*c*_, where *N*_*c*_ is the number of children in cohort *c*. For each child *j* in cohort *c*, we observe categorical items *X*_*ijc*_ with *i*=1,…, 11 corresponding to the eleven maternal reports in Table 2. Following the EFA results above, we specify two models: one in which we assume that each child is characterised by only one latent skills vector, and another in which we assume that each child is characterised by a latent bi-dimensional vector of externalising and internalising socio-emotional skills *θ*_*jc*_ = (*θ*_*jc*_^*EXT*^, *θ*_*jc*_^*INT*^).

Children are assumed to have a latent continuous propensity *X*_*ijc*_^∗^ for each item *i*=1,…, *I*. We model this propensity as a function of item- and cohort-specific intercepts *ν*_*ic*_ and loadings *λ*_*ic*_, and the child's latent skills *θ*_*jc*_, plus an independent error component *u*_*ijc*_. The propensity for each item can be written as follows:

$$X_{\mathrm{ijc}}^{*}=\nu_{\mathrm{ic}}+\lambda_{\mathrm{ic}}\theta_{\mathrm{jc}}+u_{\mathrm{ijc}}\;\text{for}\;i=1,\ldots ,11$$

or more compactly:

(4.1) $$X_{\mathrm{jc}}^{*}=\nu_{c}+\Lambda_{c}\theta_{\mathrm{jc}}+u_{\mathrm{jc}}$$

We make the common assumption of a dedicated (or congeneric) factor structure, where each measure is assumed to load on only one latent dimension (Heckman et al., 2013; Conti et al., 2010; Attanasio et al., 2018). We mirror the structure found in the exploratory factor analysis above, and assume that all items load on one factor for the 1-factor solution (Table A5), and that items 1–6 load exclusively on the externalising factor and items 7–11 on the internalising factor for the 2-factor solution (Table A6).^11^

The discrete ordered nature of the observed measures *X*_*ijc*_ is incorporated by introducing item- and cohort-specific threshold parameters *τ*_*ic*_ (Muthén, 1984). The observed measures as a function of the propensities *X*^∗^ can be then written as follows:

(4.2) $$X_{\mathrm{ijc}}=s\;\text{if}\;\tau_{s,\mathrm{ic}}\leq X_{\mathrm{ijc}}^{*}<\tau_{s+1,\mathrm{ic}}\;\text{for}\;s=0,1,2$$

with *τ*_0, *ic*_ =  − ∞ and *τ*_3, *ic*_ =  + ∞. Notice that we recode all ordered items to have higher values for *better* behaviours, so that our latent vectors can be interpreted as favourable skills and not behavioural problems.^12^

## 5 Measurement invariance

### 5.1 The configural model

Measurement invariance analysis necessarily starts from a minimally restrictive model, denominated *configural* model. This is a ‘minimum’ identifiable model, in that it places the least possible restrictions on how parameters are allowed to vary across cohorts. The restrictions implied in Eqs. (4.1), (4.2) are not sufficient to identify the parameters of the model: even with these assumptions, there are infinite equivalent parameterisations (or rotations) that deliver a minimally restrictive configural model. This is the well-known issue of factor indeterminacy, which arises due to the lack of natural units of measurement for the latent factors being assessed.

Further sets of restrictions are thus required to set the location and scale of the latent factors. Among the most straightforward and widely used parameterisations for the configural model are:

◊ Delta parameterisation [WEΔ] (Wu and Estabrook, 2016)

For all groups:

diag(Φ)=I,κ=0,ν=0,anddiag(Σ)=I.

◊ Theta parameterisation [WE*Θ*] (Wu and Estabrook, 2016)

For all groups:

(5.1) diag(Φ)=I,κ=0,ν=0,anddiag(Ψ)=I.

◊ Anchored parameterisation [MT] (Millsap and Yun-Tein, 2004)

– For all groups, normalise a reference loading to 1 for each factor.

– Set invariant across groups one threshold per item (e.g. *τ*_0, *Ai*_ = *τ*_0, *Bi*_ for two groups *A* and *B*), and an additional threshold in the reference items above.

– In the first group: *κ*_*A*_ = **0**, diag(*Σ*_*A*_) = *I*.

– Set all intercepts *ν* to zero.

The first two parameterisations (WEΔ and WE*Θ*) normalise the mean and variance of factors to the same constants in both groups, and they leave all loadings and thresholds to be freely estimated; they only differ in whether the additional required normalisation is imposed on the variances of the error terms (*Ψ*) or on the diagonal of the covariance matrix of the measures (*Σ*). The MT parameterisation instead proceeds by identifying parameters in one group first, and then imposing cross-group equality constraints to identify parameters in other groups (Wu and Estabrook, 2016). Still, all of these parameterisations are statistically equivalent. The measurement invariance analysis in this paper is based on the Theta parameterisation (WE*Θ*), but results are independent on this choice. The restrictions in Eqs. (4.1), (4.2), (5.1) define the so-called *configural* model.

### 5.2 Nested models

Any comparison between socio-emotional skills across the two cohorts requires that the measures at our disposal have the same relationship with the latent constructs of interest in both cohorts. In other words, the items in our new scale must measure socio-emotional skills in the same way in the BCS and MCS data. This property is denominated measurement invariance (MI) (Vandenberg and Lance, 2000; Putnick and Bornstein, 2016).

In the framework of factor analysis, measurement invariance is a formally testable property. In this paper, we follow the recent identification methodology by Wu and Estabrook (2016). The configural model defined above in Section 5.1 serves as the starting point. Measurement invariance is then assessed by comparing the configural model to a series of hierarchically nested models. These models place increasing restrictions on the item parameters, constraining them to be equal across groups. Their fit is then compared to that of the configural model. Intuitively, if the additional cross-group restrictions have not significantly worsened model fit, one can conclude that a certain level of invariance is achieved.

In the case where the available measures are continuous, MI analysis is straightforward (van de Schoot et al., 2012). The hierarchy of the nested models usually proceeds by testing loadings first, and then intercepts (to establish *metric* and *scalar* invariance – see Vandenberg and Lance, 2000). Invariance of systems with categorical measures, such as the scale we examine in this paper, is less well understood. In particular, the lack of explicit location and scale in the measures introduces an additional set of parameters compared to the continuous case (thresholds *τ*). This makes identification reliant on more stringent normalisations. A first comprehensive approach for categorical measures was proposed by Millsap and Yun-Tein (2004). New identification results in Wu and Estabrook (2016) indicate that, in the categorical case, invariance properties cannot be examined by simply restricting one set of parameters at a time. This is because the identification conditions used in the configural baseline model, while being minimally restrictive on their own, become binding once certain additional restrictions are imposed. In light of this, they propose models that identify structures of different invariance levels. They find that some restrictions cannot be tested alone against the configural model, because the models they generate are statistically equivalent. This is true of loading invariance, and also of threshold invariance in the case when the number of categories of each ordinal item is 3 or less. Furthermore, they suggest that comparison of both latent means and variances requires invariance in loadings, thresholds, and intercepts. A summary of the approach by Wu and Estabrook (2016) is available in Table 3.

**Table 3:** Parameterisations for measurement invariance.

Invariance level	Description	Restrictions
Configural (WE*Θ*)	• Minimally restrictive model for identification	$\text{For}\;\mathrm{all}\;\text{groups}:|\begin{array}{l} \mathrm{diag}(\Phi )=I\\ \kappa =0\\ \nu =0\\ \mathrm{diag}(\Psi )=I \end{array}$
Threshold invariance	• Restricts thresholds *τ* to be equal across groups • Statistically equivalent to configural (when measures have 3 categories or less)	*τ*_1, *ci*_ = *τ*_1, *c*′*i*_ for all items, ∀*c*, *c*′ *τ*_2, *ci*_ = *τ*_2, *c*′*i*_ for non-binary items, ∀*c*, *c*′ $\text{For}\;\mathrm{all}\;\text{groups}:|\begin{array}{l} \mathrm{diag}(\Phi )=I\\ \kappa =0 \end{array}$ $\text{For}\;\mathrm{ref}.\text{group}\;A:|\begin{array}{l} \nu_{A}=0\\ \mathrm{diag}(\Sigma_{A})=I \end{array}$
Threshold and loading invariance	• Restricts thresholds *τ* and loadings *λ* to be equal across groups • Allows comparison of latent factor variances	*τ*_1, *ci*_ = *τ*_1, *c*′*i*_ for all items, ∀*c*, *c*′ *τ*_2, *ci*_ = *τ*_2, *c*′*i*_ for non-binary items, ∀*c*, *c*′ *λ*_*ci*_ = *λ*_*c*′*i*_ for all items, ∀*c*, *c*′ For all groups: *κ* = **0** $\text{For}\;\mathrm{ref}.\text{group}\;A:|\begin{array}{l} \nu_{A}=0\\ \mathrm{diag}(\Sigma_{A})=I\\ \mathrm{diag}(\Phi_{A})=I \end{array}$
Threshold, Loading, and Intercept invariance	• Restricts thresholds *τ* and loadings *λ* to be equal across groups • Restricts intercepts *ν* to zero in both groups • Allows comparison of latent factor variances *and* means	*τ*_1, *ci*_ = *τ*_1, *c*′*i*_ for all items, ∀*c*, *c*′ *τ*_2, *ci*_ = *τ*_2, *c*′*i*_ for non-binary items, ∀*c*, *c*′ *λ*_*ci*_ = *λ*_*c*′*i*_ for all items, ∀*c*, *c*′ For all groups: *ν* = **0** $\text{For}\;\mathrm{ref}.\text{group}\;A:|\begin{array}{l} \kappa_{A}=0\\ \mathrm{diag}(\Sigma_{A})=I\\ \mathrm{diag}(\Phi_{A})=I \end{array}$

*Notes*: Adapted from Wu and Estabrook (2016).

Let's consider examples from our application. A *loading and threshold invariance* model restricts every item's loading *λ* and threshold *τ* parameters to have the same value in the two cohorts. It assumes that the items in our scale have the same relationship with latent skills across the two cohorts. In other words, items have the same salience, or informational content relative to skills. If this model fits as well as the configural model, we can be confident that the socio-emotional skills of children in the two cohorts can be placed on the same scale, and their *variances* can be compared. To see why, consider Eq. (4.1). If the loading matrix *Λ* is the same across cohorts, any difference in latent skills *Δθ* will correspond to the same difference in latent propensities *ΔX*^∗^. Equality of thresholds *τ* ensures that propensities *X*^∗^ map into observed items *X* in the same way.

A *loading, threshold, and intercept invariance* model additionally restricts every item's intercept *ν* across cohorts. A good relative fit of this model indicates that socio-emotional skills can be compared across cohorts in terms of their *means* as well. To see why, consider the following. Since the *λ* and *ν* parameters are the same across cohorts, a child in the BCS cohort with a given level of latent skills $\bar{\theta }$ will have the same expected latent item propensities *X*^∗^ as a child with the same skills in the MCS cohort. Again, equality of thresholds *τ* fixes the mapping between *X*^∗^ and *X*.^13^

We estimate the sequence of models detailed in Table 3 by mean- and variance-adjusted weighted least squares (WLSMV) – see Muthen et al. (1997); estimation starts from the items' polychoric correlation matrix, uses diagonally weighted least squares (DWLS), and exploits the full weight matrix to compute robust standard errors and test statistics. Robust WLS has proved in simulation studies to be moderately robust to small violations of the normality assumption in the latent underlying measures (Flora and Curran, 2004), and generally outperforms maximum likelihood in large samples (Beauducel and Herzberg, 2006; Li, 2016).^14^ For the purposes of the analysis, we define groups *c* as cohort-gender cells, with the reference group being males in the BCS cohort. We then compare the fit of each model against the configural model.

### 5.3 Measurement invariance results

Comparison of *χ*^2^ values across models is a common likelihood-based strategy. However, tests based on *Δχ*^2^ are known to display high Type I error rates with large sample size and more complex models such as our own (Sass et al., 2014). In fact, for all invariance levels in our applications a chi-squared difference would point to a lack of measurement invariance. The use of approximate fit indices (AFIs) is therefore recommended alongside *χ*^2^. While these indices successfully adjust for model complexity (Cheung and Rensvold, 2002), they do not have a known sampling distribution. This makes it necessary to rely on simulation studies, which derive rules of thumb indicating what level of ΔAFI is compatible with invariance.

Again, just like in the broader context of measurement invariance, most evidence regarding the performance of AFIs pertains to scenarios with continuous measures. The root mean squared error of approximation (RMSE) and the Tucker-Lewis index (TLI) are traditionally the most used AFIs in empirical practice. Simulation evidence by Cheung and Rensvold (2002) shows that these indices can show correlation between overall and relative fit, and suggest relying on additional indices, such as the comparative fit index (CFI, Bentler, 1990), McDonald non-centrality index (MFI, McDonald, 1989), and Gamma-hat index (Steiger, 1989). Subsequent simulation studies – e.g. Chen (2007) and Meade et al. (2008) – have updated these thresholds for the continuous case. In particular, Chen (2007) shows in two Monte Carlo studies that the standardised root mean square residual (RMSR) is more sensitive to lack of invariance in factor loadings than in intercepts or residual variances, while the CFI and RMSEA are equally sensitive to all three types of lack of invariance; he suggests the following thresholds for rejecting measurement invariance: ΔRMSE >.015, ΔCFI < − .010, ΔRMSR >.010.

However, it is not advisable to directly extrapolate rules of thumb derived from simulations with continuous measures to the categorical case (Lubke and Muthén, 2004). Recent studies have advanced the simulation-based evidence on the performance of AFIs in measurement invariance analysis with categorical measures. Sass et al. (2014) find that the cutoffs from Chen (2007) might not generalise well to problems estimated by WLSMV, but this is mostly confined to smaller sample sizes and detection of small degrees of non-invariance. More recently, Rutkowski and Svetina (2017) find that a ΔRMSE threshold of .010 is appropriate for testing equality of slopes and thresholds when the sample size is large, like in our case.

In any case, we present a range of fit indices to provide a more complete assessment of measurement invariance. We present the measurement invariance results for the 1-factor model in Table A10, and those for the 2-factor model in Table A11. First, by comparing the fit of each nested model across the 1-factor and the 2-factor models, it is clear that the 1-factor model fits the data significantly worse than the 2-factor model, according to all the criteria considered.^15^ Hence, in our analysis since now on, we adopt the two-factor solution, which is also consistent with the child psychology literature cited above: as mentioned above, we name the two factors externalising and internalising skills. We now examine the measurement invariance properties of our chosen two-factor solution in greater details. Looking at Panel A of Table A11, we see that the overall fit of the configural model for the chosen 2-factor solution is satisfactory according to all indices, with CFI around .95 and RMSE just above .05. As expected, given our large sample size, *χ*^2^-based tests reject measurement invariance at all levels. The model with restricted thresholds and loadings exhibits a comparable fit to the configural model, according to all the AFIs. In particular, the AFIs fall within the ranges suggested in Chen (2007), Rutkowski and Svetina (2017) and Svetina and Rutkowski (2017); see also Svetina et al. (2019) for a review of updated guidelines for measurement invariance. Invariance of loadings and thresholds across cohorts implies that the items in our scale are equally salient in their informational content, and that the latent propensities have equal mapping into the observed items.

However, further restricting intercepts results in a model where invariance is rejected across the board. In other words, intercept parameters in our model (*ν*) are estimated to be different between maternal reports in the British and Millennium Cohort Studies. This means that, for a given level of latent skills, mothers in MCS tend to assess behaviours differently from mothers in BCS. Thus, cohort differences in scores on our scale cannot be unequivocally interpreted as differences in the underlying skills, since they might also reflect differences in reporting.^16^

This is an important finding, which has to our knowledge never been acknowledged in the economic literature. How can this lack of comparability be explained? A possible interpretation is connected with secular evolution of social and cultural norms about child behaviours. For example, commonly held views of what constitutes a restless, distracted, or unhappy child might have changed between 1975 and 2006.^17^

To summarise, our measurement invariance analysis shows partial comparability of socio-emotional skills across cohorts. In particular, the variance of skills can be compared across cohorts, but mean cohort differences do not necessarily reflect differences in skills. We can use scores from our scale to compare children within the same cohort-gender group, but not across cohorts. However, we can also compare within-cohort differences between groups of children, across cohorts. As an example, consider two groups of children A and B in the BCS cohort, and two groups of children C and D in the MCS. We cannot compare the mean level of skills between groups A and C, but we can compare the mean difference between groups A and B with the mean difference between groups C and D. This is the approach we take for the rest of the paper. Refraining from direct cross-cohort comparisons, we interpreting significance and magnitude of within-cohort differences across the cohorts.

## 6 Results

**Fig. 1:**
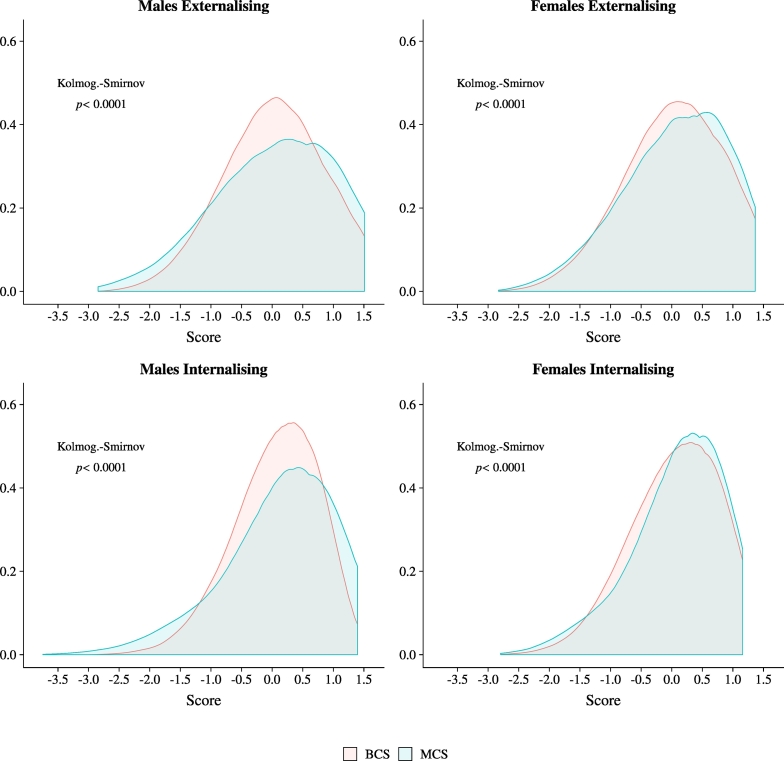
Distribution of factor scores. *Notes*: The figure shows the distribution of the externalising and internalising socio-emotional skills scores at age five obtained from the factor model, by gender and cohort. The scores are estimated from the parameter estimates in Table A13, using an Empirical Bayes Modal approach. Higher scores correspond to *better* skills. The distribution is estimated nonparametrically, using an Epanechnikov kernel. The figure also reports the *p*-value from Kolmogorov-Smirnov tests of equality between the distribution in BCS and MCS.

**Fig. A1:**
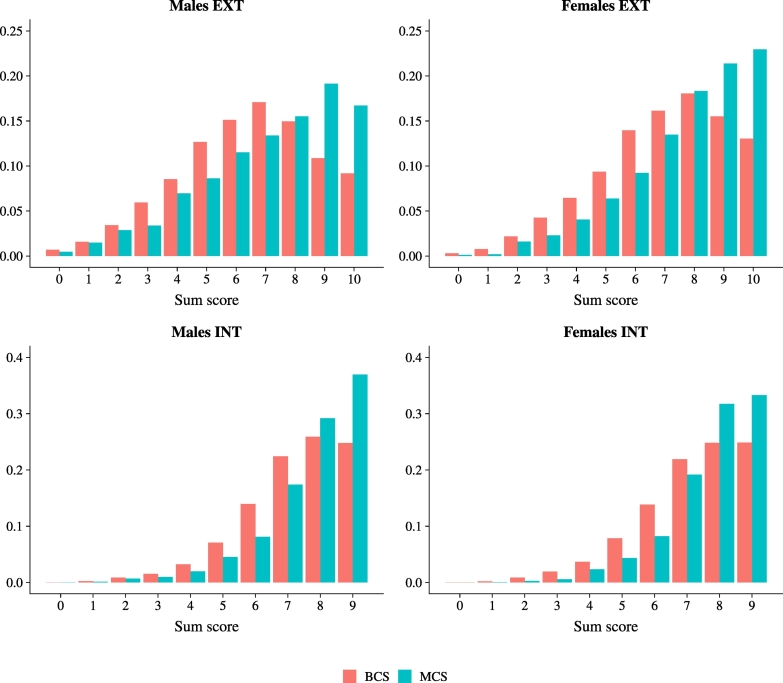
Distribution of sum scores. *Notes*: The figure shows the distribution of the externalising and internalising sum scores at age five, by gender and cohort. The scores are obtained by assigning 0, 1, or 2 points for each item in the scale in Table 2. Zero points are assigned for ‘Certainly Applies /True’ responses, one point for ‘Sometimes applies/somewhat true’, and two points for ‘Doesn't apply’. Only 0 or 1 points are assigned for items that are coded as having two categories (5 and 11). Higher scores correspond to *better* skills.

Parameter estimates from our factor model are presented in Table A13. As discussed in the previous section, loadings and thresholds are constrained to have the same value across groups. Intercepts are normalised to zero, and error variances to one, for the reference group – males in the BCS cohort. We use the estimates from this model to predict a score for each child in our sample along the latent externalising and internalising socio-emotional skill dimensions.^18^ We plot the distribution of the scores in Fig. 1. The unit of measurement is standard deviations of the distribution in the subsample of males in the BCS. Given our measurement invariance results in Section 5, we stress that the *location* of these scores should not be directly compared across cohorts. However, the shape of the distribution can be given a cross-cohort interpretation. This result is in sharp contrast with what is shown by the simple distribution of sum scores in Fig. A1: using raw scores we see an increase in mass only at the top of the distribution, while the factor scores clearly show that there is more mass in both tails of the distribution of the 2000 than of the 1970 cohort.

### 6.1 Inequality in socio-emotional skills

We find that, both unconditionally and for specific groups, inequality in socio-emotional skills at age five has increased between 1975 and 2005/6. Table 4 shows unconditional inequality statistics, using quantile differences in the distribution of skills by gender and cohort. With the exception of internalising skills in female children, all distributions have widened substantially between the BCS and MCS cohorts. The gap for both externalising and internalising skills between the 90th and the 10th percentiles for males has increased by approximately half a standard deviation. The increase in the gap is more pronounced in the bottom half of the distribution. For females, we see a narrowing at the top (90–50), but a widening at the bottom (50–10) of the distribution, again for both externalising and internalising skills. The fact that the increase in inequality is greater for the boys is consistent with the literature, which has shown that boys are more sensitive than girls to disadvantaged early environment, the presence of the mother and the quality of parenting (see for example Bertrand and Pan, 2013, and Figlio et al., 2019).

**Table 4:** Quantile differences in scores.

	Males		Females	
Quantile diff.	BCS (1970)	MCS (2000/1)	BCS (1970)	MCS (2000/1)
Externalising				

50–10	1.076	1.327	1.071	1.194
	[1.075,1.079]	[1.313,1.336]	[1.068,1.082]	[1.192,1.213]
75–25	1.081	1.373	1.123	1.164
	[1.080,1.081]	[1.360,1.390]	[1.108,1.138]	[1.151,1.179]
90–10	2.079	2.480	2.092	2.129
	[2.079,2.082]	[2.459,2.494]	[2.087,2.131]	[2.126,2.154]
90–50	1.003	1.153	1.022	0.936
	[1.000,1.003]	[1.136,1.165]	[1.018,1.053]	[0.934,0.940]
Internalising				
50–10	0.972	1.366	1.037	1.148
	[0.971,0.974]	[1.342,1.391]	[1.033,1.040]	[1.124,1.164]
75–25	0.917	1.091	1.014	0.906
	[0.916,0.919]	[1.085,1.097]	[1.011,1.018]	[0.905,0.910]
90–10	1.708	2.226	1.860	1.853
	[1.705,1.709]	[2.200,2.258]	[1.850,1.886]	[1.830,1.874]
90–50	0.735	0.859	0.823	0.706
	[0.733,0.736]	[0.858,0.882]	[0.814,0.848]	[0.706,0.711]

*Notes*: The table shows differences between quantiles of the distributions of socio-emotional skills, by gender and cohort. Bootstrap confidence intervals with 1000 repetitions are in brackets. The factor scores for socio-emotional skills are estimated using an empirical Bayes modal approach, using the parameter estimates from the factor model in Table A13. These distributions are shown in Fig. 1.

**Fig. 2:**
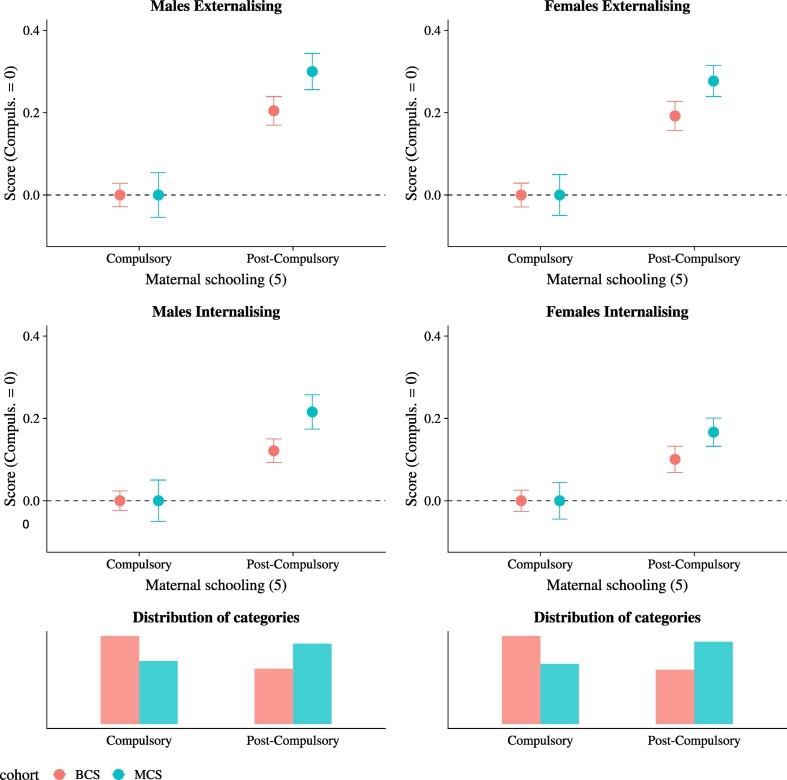
Skill inequality by mother's education. *Notes*: The figure shows unconditional mean values of socio-emotional skills scores by gender, cohort, and mother's education at age five. Mother's education is a dummy for whether the mother continued schooling past the minimum leaving age, based on her date of birth. The four panels on top present mean and 95% confidence intervals. Given that we cannot compare means of skills, all scores are normalised to take value zero for the ‘Compulsory’ category, so that the gradient is emphasised. The bottom two panels present the unconditional distribution of mother's education.

Inequality has also increased conditional on socioeconomic status. Fig. 2 shows mean skills by maternal education. We compare mothers who continued education with mothers who left school at the minimum compulsory leaving age, according to their year of birth. Given lack of comparability in the level of skills across cohort, we normalise the mean in the ‘Compulsory’ group to zero for both cohorts. For both males and females, and for both externalising and internalising skills, the difference in the socio-emotional skills of their children between more and less educated mothers has increased. The size of the increase is around .1 to .15 of a standard deviation. The increase is particularly pronounced for males, for whom it goes from .20 to .30 for externalising and from .12 to .24 for internalising.

**Fig. 3:**
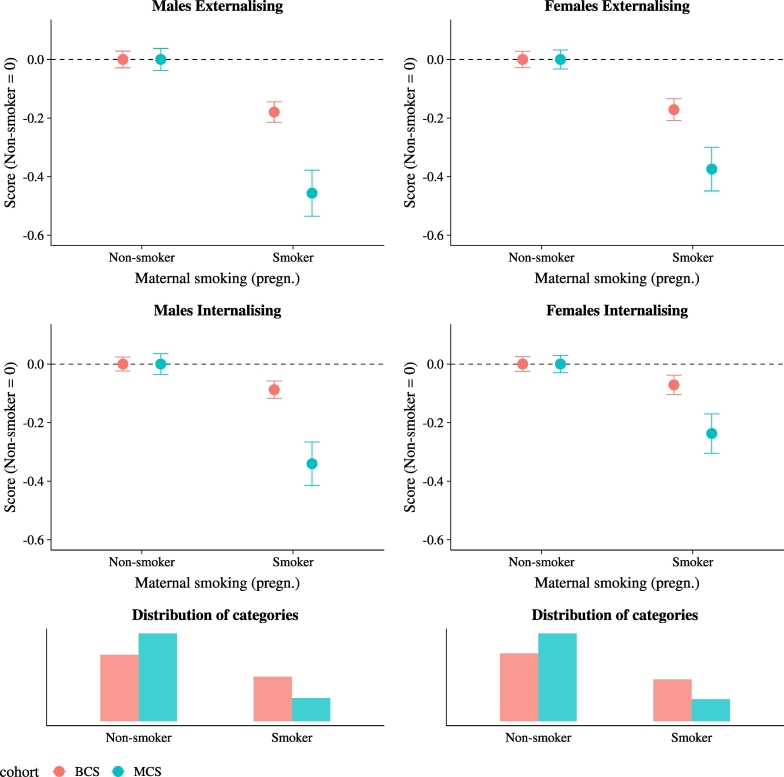
Skill inequality by mother's pregnancy smoking. *Notes*: The figure shows unconditional mean values of socio-emotional skills scores by gender, cohort, and mother's pregnancy smoking. Maternal smoking is a dummy for whether the mother reported smoking during pregnancy. The four panels on top present mean and 95% confidence intervals. Given that we cannot compare means of skills, all scores are normalised to take value zero for the ‘Non-smoker’ category, so that the gradient is emphasised. The bottom two panels present the unconditional distribution of mother smoking status in pregnancy.

**Fig. 4:**
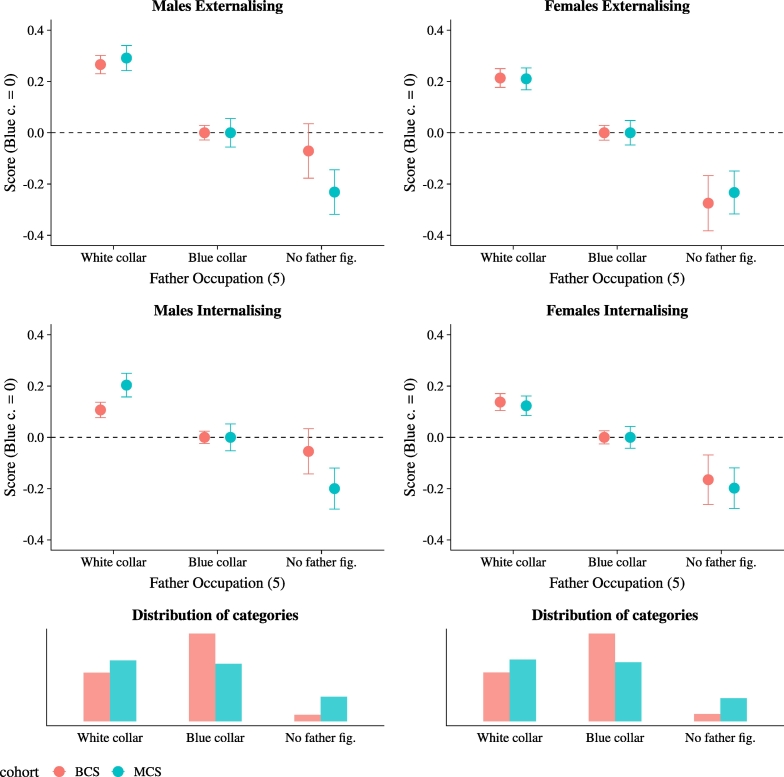
Skill inequality by father's occupation. *Notes*: The figure shows unconditional mean values of socio-emotional skills scores by gender, cohort, and father's occupation at age five. Father's occupation is based on the Registrar General's social class, with classes I to III Non Manual being ‘White collar’ and classes III Manual to V (plus ‘other’) being ‘Blue collar’. ‘No father figure’ is defined as absence of a male figure living in the household. The four panels on top present mean and 95% confidence intervals. Given that we cannot compare means of skills, all scores are normalised to take value zero for the ‘Blue collar’ category, so that the gradient is emphasised. The bottom two panels present the unconditional distribution of father's occupation. (For interpretation of the references to colour in this figure legend, the reader is referred to the web version of this article.)

Fig. 3 shows an even starker pattern when comparing children of mothers who smoked in pregnancy with non-smoking mothers. The fact that maternal smoking during pregnancy is a risk factor for offspring behavioural problems is well known in the medical literature (Gaysina et al., 2013); there is less evidence, however, on whether and to which extent these associations have changed across cohorts. The difference in child skills has increased, from less than .2 to around .4 of a standard deviation, again with the biggest increase experienced by the boys. There is also a significant increase in the gradient by paternal occupation based on social class (Fig. 4), although this is less pronounced if compared to the one based on maternal characteristics. In particular, male children with no father figure living in their household have worse skills (both internalising and externalising) compared to children with blue collar fathers in the MCS cohort. Otherwise, skill differences in father's occupation are mostly constant across the two cohorts.^19^ These patterns are in stark contrast with the findings of Reardon and Portilla (2016) for the US, who have found a narrowing of the readiness gaps from 1998 to 2010 (however, they have not tested for measurement invariance).

We then examine the same patterns as in the previous figures, but conditional on other family background indicators. The aim is to disentangle the relative contribution of each indicator to socio-emotional skills, and how it has changed in the thirty years between the two cohorts. Table 5 shows coefficients from linear regressions of socio-emotional skills at five on contemporaneous and past socioeconomic indicators, by cohort and gender. Coefficients for indicators in BCS and MCS are presented side by side, together with the *p*-value of the hypothesis that coefficients are the same in the two cohorts.^20^

**Table 5:** Determinants of socio-emotional skills across the two British cohorts.

	Externalising						Internalising					
	Males			Females			Males			Females		
	(1)	(2)	(3)	(4)	(5)	(6)	(7)	(8)	(9)	(10)	(11)	(12)
	BCS	MCS	p-value	BCS	MCS	p-value	BCS	MCS	p-value	BCS	MCS	p-value
Maternal education (5)												
Post-compulsory	0.089^∗∗∗^	0.114^∗∗∗^	[0.576]	0.099^∗∗∗^	0.142^∗∗∗^	[0.313]	0.072^∗∗∗^	0.084^∗∗^	[0.766]	0.048^∗∗^	0.081^∗∗∗^	[0.388]
	(0.027)	(0.038)		(0.027)	(0.034)		(0.022)	(0.037)		(0.024)	(0.032)	
Maternal employment (5)												
Employed	0.018	0.131^∗∗∗^	[0.008]	−0.009	0.109^∗∗∗^	[0.003]	0.041^∗∗^	0.166^∗∗∗^	[0.001]	0.031	0.155^∗∗∗^	[0.001]
	(0.024)	(0.040)		(0.024)	(0.035)		(0.020)	(0.036)		(0.022)	(0.032)	
Father occ. (5) - White collar = 0												
Blue collar	−0.195^∗∗∗^	−0.128^∗∗∗^	[0.143]	−0.118^∗∗∗^	−0.055	[0.153]	−0.076^∗∗∗^	−0.081^∗∗^	[0.901]	−0.101^∗∗∗^	−0.025	[0.052]
	(0.027)	(0.041)		(0.027)	(0.035)		(0.023)	(0.040)		(0.024)	(0.032)	
No father figure	−0.280^∗∗∗^	−0.223^∗∗∗^	[0.490]	−0.381^∗∗∗^	−0.159^∗∗∗^	[0.004]	−0.201^∗∗∗^	−0.176^∗∗∗^	[0.734]	−0.245^∗∗∗^	−0.149^∗∗∗^	[0.168]
	(0.062)	(0.059)		(0.061)	(0.052)		(0.052)	(0.054)		(0.054)	(0.049)	
Maternal background (0)												
Age	0.014^∗∗∗^	0.013^∗∗∗^	[0.792]	0.014^∗∗∗^	0.014^∗∗∗^	[0.849]	0.008^∗∗∗^	0.013^∗∗∗^	[0.135]	0.009^∗∗∗^	0.007^∗∗^	[0.612]
	(0.002)	(0.004)		(0.003)	(0.003)		(0.002)	(0.004)		(0.002)	(0.003)	
Unmarried	0.065	−0.122^∗∗∗^	[0.009]	0.025	−0.140^∗∗∗^	[0.013]	0.114^∗∗^	−0.028	[0.025]	0.035	−0.055^∗^	[0.133]
	(0.057)	(0.043)		(0.059)	(0.038)		(0.049)	(0.040)		(0.051)	(0.034)	
Nonwhite child	−0.161^∗∗^	−0.231^∗∗∗^	[0.461]	−0.029	−0.222^∗∗∗^	[0.020]	0.025	−0.125^∗∗^	[0.078]	0.080	−0.166^∗∗∗^	[0.001]
	(0.082)	(0.064)		(0.069)	(0.044)		(0.070)	(0.055)		(0.060)	(0.042)	
Pregnancy												
Firstborn	−0.121^∗∗∗^	−0.011	[0.023]	−0.070^∗∗^	0.037	[0.021]	−0.186^∗∗∗^	−0.087^∗∗^	[0.021]	−0.161^∗∗∗^	−0.035	[0.003]
	(0.030)	(0.044)		(0.029)	(0.038)		(0.025)	(0.040)		(0.026)	(0.034)	
Mother smoked in pregnancy	−0.145^∗∗∗^	−0.233^∗∗∗^	[0.077]	−0.110^∗∗∗^	−0.155^∗∗∗^	[0.360]	−0.077^∗∗∗^	−0.165^∗∗∗^	[0.048]	−0.036	−0.108^∗∗∗^	[0.103]
	(0.025)	(0.050)		(0.025)	(0.044)		(0.021)	(0.045)		(0.022)	(0.039)	
(log) Birthweight	0.146^∗∗^	0.308^∗∗∗^	[0.191]	0.186^∗∗^	0.287^∗∗∗^	[0.392]	0.095	0.272^∗∗^	[0.108]	0.123^∗^	0.057	[0.535]
	(0.076)	(0.113)		(0.081)	(0.096)		(0.060)	(0.109)		(0.072)	(0.086)	
Adj. R^2^	0.062	0.094		0.056	0.100		0.042	0.071		0.042	0.061	
Num. obs.	4565	2799		4313	2701		4565	2799		4313	2701	

*Notes*: The table shows coefficients from linear regressions of children's socio-emotional skills at five years of age on family background characteristics. The dependent variable is a factor score obtained from the factor model in Section 4. Col. (1) and (2) show coefficients and standard errors in parentheses, for male children in the BCS and MCS cohorts separately. The latter are obtained using 1000 bootstrap repetitions, taking into account the factor estimation stage that precedes the regression. Col. (3) shows the *p*-value of a test that the coefficient is the same in the two cohorts. Col. (4) to (6) repeat for female children. Col. (7) to (12) repeat for internalising skills. All estimates additionally control for region of birth, mother height, number of previous stillbirths at child's birth, preterm birth, a dummy for missing gestational age, and number of other children in the household at child age 5. See Table A1 for a description of the variables used. ^∗∗∗^ p≤0.01, ^∗∗^ p≤0.05, ^∗^ p≤0.1.

Overall, the importance of maternal socioeconomic status (education and in particular employment) in determining socio-emotional skills has increased from the BCS to the MCS children. The ‘premium’ in skills for children of better educated and employed mothers is significantly larger, for both boys and girls, internalising and externalising skills. At the same time, the penalty for having a blue-collar father, or not having a father figure at all in the household, has significantly declined across the two cohorts, especially for girls. Being born to an unmarried mother, and to a mother who smoked during pregnancy, is associated with a higher penalty for both dimensions of socio-emotional skills in the latter cohort.^21^ Children of non-white ethnicity have worse internalising and externalising skills in the MCS, a penalty almost absent in the BCS (where the prevalence of non-white children was much lower). Firstborn boys and girls in the BCS have worse skills, but this difference disappears in the MCS. Lastly, we document an increase in the returns to birth weight, which is more pronounced for boys.

These changes in the relative importance of pregnancy factors and family background characteristics for child socio-emotional skills at age 5 need to be interpreted in the light of the significant changes in the prevalence of such characteristics across cohorts. As shown in Table 1, the age of the mother at birth, and the proportion of mothers non-smoking in pregnancy, with post-compulsory education and in employment at the age 5 of the child has substantially increased; at the same time, the proportion of households with no father figure has increased, and so the proportion of women unmarried at birth is much higher in the 2000 than in the 1970 cohort. Also, as noted, the ethnic structure of the population has changed, with a higher proportion of non-white children in the MCS than in the BCS. In general, this has been a period of significant societal changes, with an almost continual rise in the proportion of women in employment, an older age at first birth and a rise in dual-earning parents families (Roantree and Vira, 2018).

Hence, we lastly attempt to disentangle whether and to which extent the observed changes in inequality in socio-emotional skills across the two cohorts can be attributed to changes in returns (or penalties) to characteristics such as maternal education, or to compositional changes. To this aim, we use the method recently developed by Firpo et al. (2018)^22^ as an extension of the Oaxaca-Blinder (OB) decomposition to any distributional measure, that here we apply for the first time to changes in inequality in early childhood development. This two-stage procedure first decomposes distributional changes into a ‘composition effect’ and a ‘coefficient effect’ using a reweighting method; then it further divides these two components into the contribution of each explanatory variable, using Recentered Influence Function (RIF) regression (Firpo et al., 2009).

Following Firpo et al. (2018), we first perform an OB decomposition using the BCS sample and the counterfactual sample (BCS reweighted to be as MCS)^23^ to get the pure composition effect, using the BCS as reference coefficients. The total unexplained effect in this decomposition corresponds to the specification error, and allows to assess the importance of departures from the linearity assumption. Second, we perform the decomposition using the MCS sample and the counterfactual sample, to obtain the pure coefficient effect (the ‘unexplained’ part); the explained effect in this decomposition corresponds to the reweighting error, which allows to assess the quality of the reweighting.

**Fig. 5:**
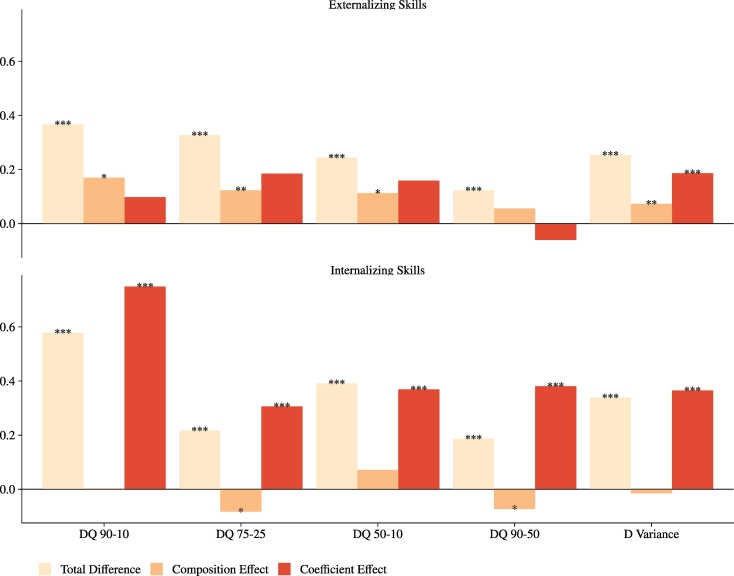
RIF decomposition of changes in measures of inequality in socio-emotional skills – Males. *Notes*: The figures show the total changes in five measures of inequality in socio-emotional skills between the BCS and the MCS, and decomposes them in composition and coefficient effects, following the RIF decomposition (with reweighting) of Firpo et al. (2018). The top figure presents the decompositions for the externalising skills score, and the bottom figure for the internalising skills score. The five inequality measures considered are the quantile differences 90–10, 75–25, 50–10, 90–50, and the variance. Full results are in Table A14 and Table A15. Bootstrapped standard errors over the entire procedure (500 replications) were used to compute the *p*-values. ^⁎⁎⁎^p ≤ 0.01, ^⁎⁎^ p ≤ 0.05, ^⁎^ p ≤ 0.1.

In Fig. 5 we present the results of the RIF decomposition for changes in five measures of inequality in socio-emotional skills for the boys, both externalising (top figure) and internalising (bottom figure). The results indicate that different factors explain the rise in inequality in the two skills: on the one hand, compositional changes explain, on average, half of the cross-cohort increase in inequality in externalising skills, regardless of the measure considered^24^; on the other hand, the increase in inequality in internalising skills seems to be entirely explained (even over-explained) by changes in returns (or penalties) to background characteristics. Composition and coefficient effects are further decomposed in the contribution of each covariate, and the results presented in Table A14 and in Table A15. We see in Table A14 that mother's age and marital status at birth are the two variables that best account for the compositional changes, driving the increase in inequality in externalising skills among the boys for the quantile differences and the variance, respectively. This is hardly surprising, given that we have seen in Table 1 that the average age of the mother at birth has increased by approximately three years (from 26 to 29 years old), and that the proportion of unmarried mothers has increased dramatically, from 5% in the BCS to 36% in the MCS. The baseline covariates, instead, do a less impressive job at explaining the changes in coefficients underlying the increase in inequality in internalising skills (Table A15, note the changes in returns to maternal employment go in the direction of reducing inequality). This can be partly explained by the fact that, due to lack of comparable measures across cohorts, we have been unable to account for important determinants of a child's internalising behaviour, such as for example maternal mental health. We also notice that, for the quantile differences 75–25 and 90–50, the composition effect is significant but negative; in other words, compositional changes linked to maternal marriage status would have led to a reduction in inequality, especially at the top of the distribution. Reassuringly, both the specification and the reweighting error are not significantly different from zero. Lastly, the results are not so clear-cut for the girls, who experienced a more muted increase in inequality, concentrated at the bottom of the distribution. The RIF results displayed in Table A16 show that no single contributing factor emerges.

### 6.2 Socio-emotional skills and adolescent/adult outcomes

In this last section, we study the predictive power of socio-emotional skills for adolescent and adult outcomes, to gain some insights as to whether inequality in the early years could translate into later life inequalities. We contribute to a vast interdisciplinary literature by examining medium- and long-term impacts of skills measured at an earlier age than in previous studies, i.e. well before the start of formal education. Showing that these early skills are predictive of different later outcomes across various domains provide a key rationale for the role of early intervention in reducing life course inequalities. In practice, we proceed by regressing health and socioeconomic outcomes measured in adolescence and adulthood on the socio-emotional skills scores at age five obtained by our factor model, controlling for the harmonised family background variables at birth and age five (see Table A1).^25^ We present results with and without controlling for cognitive skills. As detailed in Section 3, the available cognitive measures are not comparable across cohorts. Still, we control for a factor score that summarises all information on cognitive skills that is available in each cohort, regardless of their comparability.

Socio-emotional skills at five years of age are predictive of adolescent health behaviour and outcomes in both cohorts.^26^ Table 6 examines adolescent smoking and BMI for both cohorts; Table A19 reports the results for the same outcomes in adulthood (at age 42), for the BCS only. Externalising skills are negatively correlated to subsequent smoking and BMI in both cohorts, for both genders. Recall that a child with high externalising skills exhibits less restless and hyperactive behaviour, and has less anti-social conduct. Our findings are consistent with the body of evidence reviewed in Section 2, which shows that better socio-emotional skills (measured using different scales and at various points during childhood and adolescence) are negatively associated with smoking. At the same time, internalising skills are positively correlated with smoking (only in the 1970 cohort) and BMI (only for girls), although less strongly than externalising skills. This apparently counterintuitive result makes sense in light of the items in our internalising scale shown in Table 2. A child with better internalising skills is less solitary, neurotic, and worried. From this perspective, he/she is likely more sociable and subject to peer influence in health behaviours. This is consistent with the evidence in Goodman et al. (2015), who find a positive association between child emotional health (measured with items from the internalising behaviour subscale of the Rutter scale at age 10 in the BCS) and smoking at age 42. Furthermore, in recent work Hsieh and van Kippersluis (2018) have shown personality to be a key mechanism through which peers affect smoking behaviour. We have also tested the robustness of these findings by jointly estimating by maximum likelihood the measurement system (with the partial invariance constraints) and the two outcome equations for smoking and BMI. The results, presented in Table A18, are qualitatively similar to those obtained with the two-step method.^27^

**Table 6:** Predictors of adolescent outcomes.

	Males			Females		
	Mean	Coefficients		Mean	Coefficients	
	(1)	(2)	(3)	(4)	(5)	(6)
*Tried smoking (BCS - 16)*	0.524			0.586		
Externalising skills (5)		−.073^∗∗∗^	−.081^∗∗∗^		−.068^∗∗∗^	−.077^∗∗∗^
		(.024)	(.025)		(.021)	(.022)
Internalising skills (5)		.055^∗∗^	.060^∗∗^		.039	.045^∗^
		(.028)	(.029)		(.025)	(.026)
Cognitive skills (5)			.010			.012
			(.019)			(.017)
Adj. R^2^		0.032	0.032		0.048	0.046
Observations		1197	1123		1693	1581
*BMI (BCS - 16)*	20.9			21.2		
Externalising skills (5)		−.178	−.227^∗^		−.225^∗^	−.222^∗^
		(.118)	(.122)		(.122)	(.128)
Internalising skills (5)		.036	.062		.280^∗∗^	.234
		(.140)	(.146)		(.141)	(.145)
Cognitive skills (5)			.021			−.093
			(.101)			(.101)
Adj. R^2^		0.017	0.018		0.023	0.023
Observations		1640	1531		1873	1757
*Tried smoking (MCS - 14)*	0.119			0.152		
Externalising skills (5)		−.027^∗∗^	−.026^∗∗^		−.016	−.014
		(.011)	(.012)		(.013)	(.013)
Internalising skills (5)		.006	.008		.010	.010
		(.012)	(.012)		(.014)	(.014)
Cognitive skills (5)			−.006			−.021^∗^
			(.010)			(.012)
Adj. R^2^		0.051	0.049		0.039	0.040
Observations		1998	1973		2025	2019
*BMI (MCS - 14)*	20.7			21.6		
Externalising skills (5)		−.262^∗∗^	−.238^∗^		−.453^∗∗∗^	−.411^∗∗∗^
		(.131)	(.133)		(.141)	(.142)
Internalising skills (5)		.033	.045		.330^∗∗^	.332^∗∗^
		(.142)	(.143)		(.158)	(.157)
Cognitive skills (5)			−.070			−.361^∗∗^
			(.122)			(.160)
Adj. R^2^		0.022	0.022		0.049	0.051
Observations		2006	1976		1937	1928

*Notes*: The table shows coefficients from linear regressions of cohort members' adolescent outcomes on their externalising and internalising socio-emotional skills at five years of age. Col. (1) shows the mean of the outcome for males. Col. (2) regresses the outcome on the scores obtained from the factor model in Section 4. Col. (3) additionally controls for cognitive ability at age five. This is a simple factor score obtained by aggregating the available cognitive measures. All standard errors in parentheses are obtained using 1000 bootstrap repetitions, taking into account the factor estimation stage that precedes the regression. Col. (4) to (6) repeat for female cohort members. All estimates additionally control for region of birth, maternal education (5), maternal employment (5), father occupation (5), maternal background (age, height, nonwhite ethnicity, number of children in the household), pregnancy (firstborn child, number of previous stillbirths, mother smoked in pregnancy, preterm birth, (log) birth weight). See Table A1 for a description of the variables used. ^∗∗∗^ p≤0.01, ^∗∗^ p≤0.05, ^∗^ p≤0.1.

Conditional on socio-emotional skills, cognition has limited predictive power for these behaviours, and only for girls.^28^ This is in line with the evidence in Conti and Heckman (2010), who show that not accounting for non-cognitive traits (in their paper, a self-regulation factor measured at age 10) overestimates the importance of cognition for predicting health and health behaviours, using data from the British cohort study. Along the same lines, Conti and Hansman (2013) use rich data on child personality and socio-emotional traits collected at ages 7, 11 and 16 in the 1958 British birth cohort,^29^ and show that these traits rival the importance of cognition in explaining the education gradient in health behaviours (including smoking and BMI). We show that child socio-emotional skills have greater predictive power than cognition for health outcomes and behaviours even when measured at an earlier age than in previous work.

Cohort members from the British Cohort Study are now well into their adulthood. For this cohort, we can examine the association between socio-emotional skills at age five and adult education and labour market outcomes. The structure of Table 7 is similar to Table 6, but it considers educational achievement, employment, and earnings (conditional on being in paid employment) for the BCS cohort members. For these outcomes, the predictive power of cognitive skills outweighs that of socio-emotional skills, which are only predictive of educational attainment, and whose predictive power for males is driven to insignificance after controlling for cognition. This is consistent with the evidence in Conti et al. (2011), who show that cognitive endowments at age 10 are more predictive (than socio-emotional and health ones) for employment and wage outcomes in the BCS. Again, we show that the greater predictive power of cognition for socioeconomic outcomes holds even when considering earlier-life measures of child development.

**Table 7:** Predictors of adult outcomes – BCS.

	Males			Females		
	Mean	Coefficients		Mean	Coefficients	
	(1)	(2)	(3)	(4)	(5)	(6)
*Higher education (34)*	.430			.426		
Externalising skills (5)		.044^∗∗^	.024		.069^∗∗∗^	.053^∗∗∗^
		(.022)	(.022)		(.020)	(.020)
Internalising skills (5)		−.032	−.026		−.017	−.029
		(.025)	(.026)		(.023)	(.023)
Cognitive skills (5)			.088^∗∗∗^			.113^∗∗∗^
			(.018)			(.016)
Adj. R^2^		0.083	0.099		0.101	0.120
Observations		1320	1237		1691	1589
*Employed (42)*	.932			.828		
Externalising skills (5)		.012	.010		.014	.014
		(.011)	(.011)		(.017)	(.018)
Internalising skills (5)		.022^∗^	.020		.024	.017
		(.013)	(.013)		(.018)	(.019)
Cognitive skills (5)			.023^∗∗^			.037^∗∗∗^
			(.010)			(.013)
Adj. R^2^		0.056	0.052		0.010	0.014
Observations		1294	1216		1677	1571
*(log) Gross weekly pay (42)*	6.474			5.775		
Externalising skills (5)		.047	.047		.009	.003
		(.037)	(.035)		(.043)	(.045)
Internalising skills (5)		−.044	−.081^∗^		.051	.041
		(.046)	(.043)		(.048)	(.050)
Cognitive skills (5)			.064^∗∗^			.137^∗∗∗^
			(.029)			(.033)
Adj. R^2^		0.057	0.068		0.046	0.061
Observations		918	865		1198	1122

*Notes*: The table shows coefficients from linear regressions of BCS cohort members' adult outcomes on their externalising and internalising socio-emotional skills at five years of age. Col. (1) shows the mean of the outcome for males. Col. (2) regresses the outcome on the scores obtained from the factor model in Section 4. Col. (3) additionally controls for cognitive ability at age five. This is a simple factor score obtained by aggregating the available cognitive measures. All standard errors in parentheses are obtained using 1000 bootstrap repetitions, taking into account the factor estimation stage that precedes the regression. Col. (4) to (6) repeat for female cohort members. All estimates additionally control for region of birth, maternal education (5), maternal employment (5), father occupation (5), maternal background (age, height, nonwhite ethnicity, number of children in the household), pregnancy (firstborn child, number of previous stillbirths, mother smoked in pregnancy, preterm birth, (log) birth weight). See Table A1 for a description of the variables used. ^∗∗∗^ p≤0.01, ^∗∗^ p≤0.05, ^∗^ p≤0.1.

## 7 Conclusion

In this paper we have studied inequality in a dimension of human capital which has received less attention than others in the literature so far: socio-emotional skills very early in life. In particular, we have focused on the measurements of these skills at age 5 in two British cohorts born 30 years apart: the one of children born in 1970 (British Cohort Study, BCS) and the one of children born in 2000/1 (Millennium Cohort Study, MCS). We have provided a timely contribution to the recent but flourishing literature on the determinants and consequences of early human development, by bridging it with the inequality literature.

We have taken very seriously the issue of comparability of measurements of socio-emotional skills across cohorts. First, we have selected 11 comparable items across two related scales: the Rutter scale in the BCS, and the Strength and Difficulties Questionnaire (SDQ) in the MCS. After examining the latent structure underlying the items, we have identified by means of exploratory factor analysis two dimensions of socio-emotional skills. We have labeled them ‘internalising’ and ‘externalising’ skills, the former related to the ability of children to focus their concentration and the latter to engage in interpersonal activities.

Second, we have formally tested for measurement invariance across the two cohorts (for each gender) of the 11 items comprising the two externalising and internalising scales, following recent methodological advances in factor analysis with categorical outcomes. We have found only partial support for measurement invariance, with the implication that we have only been able to compare how inequality in these socio-emotional skills has changed across the two cohorts, but not whether their average level is higher or lower in one of them. These results sound a warning to research in this area which routinely compares levels of skills across different groups (at different times, or of different gender), without first establishing their comparability.

Third, after having computed comparable scores for both externalising and internalising skills, and for both boys and girls, we have compared how inequality in these skills has changed across the 1970 and the 2000 cohorts. We have documented for the first time that inequality in these early skills has increased, especially for boys. The cross-cohort increase in the gap is more pronounced at the bottom of the distribution (50–10 percentiles). We have also documented changes in conditional skills gaps across cohorts. In particular, the difference in the socio-emotional skills of their children between mothers of higher and lower socio-economic status (education and employment) has increased. The increase in cross-cohort inequality is even starker when comparing children born to mothers who smoked during pregnancy. On the other hand, the skills penalty arising from the lack of a father figure in the household has substantially declined. Moreover, we have formally decomposed the increase in inequality into compositional changes, and changes in returns to maternal characteristics - providing the first child development application of the method recently developed by Firpo et al. (2018). We have found that half of the increase in inequality in externalising skills across cohorts can be explained by compositional changes, with maternal age and marital status at birth being the most important factors; on the other hand, the increase in inequality in internalising skills seems to be entirely driven by changes in returns to maternal characteristics.

Fourth, we have contributed to the literature on the predictive power of socio-emotional skills by showing that even skills measured at a much earlier age than in previous work are significantly associated with outcomes both in adolescence and adulthood. In particular, socio-emotional skills are more significant predictors of health and health behaviours (smoking and BMI), while cognition has greater predictive power for socioeconomic outcomes (education, employment and wages). Our results ultimately show the importance of inequalities in the early years development for the accumulation of health and human capital across the life course.

## Figures and Tables

**Table A1:** Description of harmonised variables.

Variable group	Age	Variable	Note
Maternal education	5	Post-compulsory schooling^*d*^	Whether mother continued schooling past the compulsory age, based on her year of birth. School leaving age in England was changed from 14 to 15 in 1947 and from 15 to 16 in 1972.
Maternal employment	5	Employed^*d*^	Includes full time and part time.
Father occupation	5	White collar (I-IIINM)^*d*^ Blue collar (IIIM-V-other)^*d*^ No father figure^*d*^	Based on father's Registrar General Social Class classification of occupations. White collar includes I (Professional), II (Managerial/technical), IIINM (Skilled non-manual). Blue collar includes IIIM (Skilled manual), IV (Partly skilled), V (Unskilled), Other, Unemployed, and Armed forces. No father figure is a dummy for children whose father does not live in the same household. Father's social class was recorded using the SOC2000 classification in MCS. We use the derivation matrices kindly provided by David Pevalin at ISER (available at https://www.iser.essex.ac.uk/archives/nssec/derivations-of-social-class) to map SOC2000 into Registrar General Social Class.
Maternal background	0/5	Mother's age at birth Mother's height (cm) Mother unmarried at birth^*d*^ Child nonwhite ethnicity^*d*^ Number of children in HH	All variables are self-reported by the mother at birth, except for number of children in household (at five years old). Unmarried is only based on marital status, and includes cohabitation.
Pregnancy	0	Child is firstborn^*d*^ Number of previous stillbirths Mother smoked in pregnancy^*d*^ Preterm birth (under 37 weeks gestation)^*d*^ (log) birth weight (kg)	Parity, stillbirths, and smoking are self-reported by the mother. Gestational length and birth weight are from hospital records.
Cognitive skills	5		Based on test batteries administered to the cohort member at five. Three tests are used for BCS children: Copy Designs (child is asked to copy simple designs adjacently), Human Figure Drawing (child draws an entire human figure), English Picture Vocabulary Test (child identifies the picture referring to a word among four pictures). Three tests are used in the MCS: BAS Naming Vocabulary (child is shown a series of pictures and asked to name it), BAS Picture Similarity (child is shown a row of 4 pictures on a page and places a card with a fifth picture under the one most similar to it), BAS Pattern Construction (child constructs a design by putting together flat squares or solid cubes with patterns on each side).
Adolescent outcomes	16 (BCS) 14 (MCS)	Child tried smoking^*d*^ Body Mass Index (BMI)	Smoking is self-reported by the child. Height and weight are taken as part of a medical examination.
Adult outcomes (BCS only)	34 42 34, 42	Higher education^*d*^ Employed^*d*^ (log) gross weekly pay	Higher education is defined as having a university degree or its vocational equivalent. It corresponds to level 4 or 5 in the National Vocational Qualification (NVQ) equivalence. Employed is a dummy for being in paid employment or self-employment, either full or part time. Gross weekly pay is weekly pre-tax pay from the respondent's main activity, conditional on being a paid employee.

*Notes*: Variables denoted by ^*d*^ are binary or categorical.

**Table A2:** Behavioural screening scales in the BCS and MCS five-year surveys.

Panel A: Rutter A Scale (Rutter et al., 1970) – British Cohort Study (1975) five-year survey
1. Very restless. Often running about or jumping up and down. Hardly ever still.^⁎^
2. Is squirmy or fidgety.^⁎^
3. Often destroys own or others' belongings.
4. Frequently fights other children.^⁎^
5. Not much liked by other children.
6. Often worried, worries about many things.^⁎^
7. Tends to do things on his/her own, is rather solitary.^⁎^
8. Irritable. Is quick to fly off the handle.
9. Often appears miserable, unhappy, tearful or distressed.^⁎^
10. Sometimes takes things belonging to others.
11. Has twitches, mannerisms or tics of the face or body.
12. Frequently sucks thumb or finger.
13. Frequently bites nails or fingers.
14. Is often disobedient.^⁎^
15. Cannot settle to anything for more than a few moments.^⁎^
16. Tends to be fearful or afraid of new things or new situations.^⁎^
17. Is over fussy or over particular.
18. Often tells lies.
19. Bullies other children.^⁎^
A. Complains of headaches.^⁎^
B. Complains of stomach-ache or has vomited.^⁎^
D. Has temper tantrums (that is, complete loss of temper with shouting, angry movements, etc.).^⁎^
Panel B: Strength and Difficulties Questionnaire (Goodman, 1997) – Millennium Cohort Study (2000/1) five-year survey
1. Considerate of other people's feelings.
2. Restless, overactive, cannot stay still for long.^⁎^
3. Often complains of head- aches, stomach-ache or sickness.^⁎^
4. Shares readily with other children (treats, toys, pencils, etc.).^+^
5. Often has temper tantrums or hot tempers.^⁎^
6. Rather solitary, tends to play alone.^⁎^
7. Generally obedient, usually does what adults request.^⁎+^
8. Many worries, often seems worried.^⁎^
9. Helpful if someone is hurt, upset or feeling ill.^+^
10. Constantly fidgeting or squirming.^⁎^
11. Has at least one good friend.^+^
12. Often fights with other children or bullies them.^⁎^
13. Often unhappy, down-hearted or tearful.^⁎^
14. Generally liked by other children.^+^
15. Easily distracted, concentration wanders.^⁎^
16. Nervous or clingy in new situations, easily loses confidence.^⁎^
17. Kind to younger children.^+^
18. Often lies or cheats.
19. Picked on or bullied by other children.
20. Often volunteers to help others (parents, teachers, other children).^+^
21. Thinks things out before acting.^+^
22. Steals from home, school or elsewhere.
23. Gets on better with adults than with other children.
24. Many fears, easily scared.
25. Sees tasks through to the end, good attention span.^+^

*Notes*: Items denoted by ^+^ are positively worded in the original scale. Items denoted by ^⁎^ are retained in the new 11-item comparable scale.

**Table A3:** Item prevalence, by cohort and gender.

				Males						Females					
				BCS			MCS			BCS			MCS		
				Cert. Appl.	Smtm. Appl.	Appl.	Cert. True	Smwt. True	True	Cert. Appl.	Smtm. Appl.	Appl.	Cert. True	Smwt. True	True
Itm.	Factor	Cat.	Title	(%)	(%)	(%)	(%)	(%)	(%)	(%)	(%)	(%)	(%)	(%)	(%)
1	EXT	3	Restless	32.1	40.4		17.5	29.1		25.0	40.4		13.3	24.1	
2	EXT	3	Squirmy/fidgety	12.3	31.8		11.3	29.4		11.3	32.1		8.9	25.4	
3	EXT	3	Fights/bullies	6.6	39.3		1.7	9.2		3.1	28.1		0.9	5.0	
4	EXT	3	Distracted	8.0	30.1		15.9	44.3		6.1	25.6		9.7	38.8	
5	EXT	2	Tantrums			26.4			51.1			19.6			46.8
6	EXT	2	Disobedient			73.7			49.0			64.9			41.6
7	INT	3	Worried	5.6	29.4		2.4	11.8		5.8	31.2		1.5	11.9	
8	INT	3	Fearful	7.0	29.2		11.0	34.6		6.6	30.0		9.8	38.0	
9	INT	3	Solitary	9.7	37.4		6.4	26.1		8.5	35.3		5.1	24.2	
10	INT	3	Unhappy	2.3	18.3		1.6	9.2		3.0	22.4		1.6	8.3	
11	INT	2	Aches			13.3			17.3			14.8			22.3

*Notes*: The table shows the prevalence by gender and cohort for each item of our novel subscale. *Itm.* is item number. *Factor* is the latent construct to which the item loads – EXT is Externalising skills, INT is Internalising skills. *Cat.* is the number of categories in which the item is coded – 2 denotes a binary item (applies/does not apply) and 3 denotes a 3-category item. *Title* is a short label for the item. *Cert./Smtm. Appl.* = Certainly/sometimes applies. *Cert./Smwt. True* = Certainly/somewhat true.

**Table A4:** Suggested number of factors to retain – 11-item scale.

	BCS (1970)			MCS (2000/1)		
Approach	All	Males	Females	All	Males	Females
Optimal coordinates	3	3	3	2	2	3
Acceleration factor	1	1	1	1	1	1
Parallel analysis	3	3	3	2	2	3
Kaiser	3	3	3	2	2	3
VSS Compl. 1	2	2	1	1	1	1
VSS Compl. 2	2	2	2	2	2	2
Velicer MAP	1	1	1	2	2	2

*Notes*: The table compares the optimal number of factors suggested by different approaches, for our novel scale: scree test based approaches (optimal coordinates, acceleration factor – Raîche et al., 2013), parallel analysis (Horn, 1965), Kaiser's eigenvalue rule (Kaiser, 1960), Very Simple Structure (VSS, Revelle and Rocklin, 1979), Velicer Minimum Average Partial test (MAP, Velicer, 1976).

**Table A5:** Loadings from exploratory factor analysis with 1 or 3 factors – 11-item scale.

		BCS (1970) - 1 Fac.		BCS (1970) - 3 Fac.			MCS (2000/1) - 1 Fac.		MCS (2000/1) - 3 Fac.		
Item	Title	Males	Females	Females Fac. 1	Females Fac. 2	Females Fac. 3	Males	Females	Females Fac. 1	Females Fac. 2	Females Fac. 3
1	Restless	**0.667**	**0.627**	**0.761**	−0.079	−0.041	**0.762**	**0.697**	**0.879**	−0.083	0.059
2	Squirmy/fidgety	**0.626**	**0.65**	**0.703**	0.094	−0.118	**0.696**	**0.669**	**0.716**	0.019	0.088
3	Fights/bullies	**0.519**	**0.492**	**0.468**	−0.068	0.176	**0.673**	**0.603**	**0.383**	**0.361**	−0.136
4	Distracted	**0.605**	**0.644**	**0.641**	0.07	−0.007	**0.64**	**0.604**	**0.607**	0.068	0.026
5	Tantrums	**0.584**	**0.557**	**0.461**	0.088	0.133	**0.633**	**0.612**	**0.409**	**0.362**	−0.18
6	Disobedient	**0.627**	**0.601**	**0.582**	−0.063	0.169	**0.533**	**0.47**	**0.46**	0.158	−0.241
7	Worried	**0.319**	**0.39**	−0.008	**0.807**	−0.009	**0.515**	**0.433**	−0.12	**0.666**	0.181
8	Fearful	0.255	0.283	−0.044	**0.56**	0.055	**0.387**	**0.311**	−0.05	**0.404**	0.144
9	Solitary	0.212	0.295	−0.009	0.028	**0.787**	**0.393**	**0.311**	0.093	0.102	**0.773**
10	Unhappy	**0.51**	**0.548**	0.291	**0.385**	0.144	**0.673**	**0.589**	0.047	**0.728**	0.025
11	Aches	**0.317**	0.278	0.104	**0.304**	0.01	**0.392**	**0.432**	0.054	**0.497**	−0.011

*Notes*: The table displays the factor loadings obtained from exploratory factor analysis (EFA) on our novel scale, separately by cohort. The EFA is performed decomposing the polychoric correlation matrix of the items and using weighted least squares, and the solution is rescaled using oblique factor rotation (*oblimin*). We use the **R** package psych, version 1.8.4 (Revelle, 2018).

**Table A6:** Loadings from exploratory factor analysis with 2 factors – 11-item scale.

		BCS (1970)		MCS (2000/1)	
Item	Title	Factor 1 (EXT)	Factor 2 (INT)	Factor 1 (EXT)	Factor 2 (INT)
1	Restless	**0.79**	−0.113	**0.912**	−0.075
2	Squirmy/fidgety	**0.67**	0.021	**0.741**	0.029
3	Fights/bullies	**0.499**	0.046	**0.49**	0.26
4	Distracted	**0.629**	0.05	**0.663**	0.035
5	Tantrums	**0.484**	0.177	**0.486**	0.209
6	Disobedient	**0.598**	0.066	**0.557**	−0.008
7	Worried	−0.037	**0.729**	−0.087	**0.777**
8	Fearful	−0.064	**0.595**	−0.031	**0.487**
9	Solitary	0.075	**0.312**	0.01	**0.455**
10	Unhappy	0.249	**0.507**	0.104	**0.768**
11	Aches	0.135	0.268	0.028	**0.463**

*Notes*: The table displays the factor loadings obtained from exploratory factor analysis (EFA) on our novel scale, separately by cohort. The EFA is performed decomposing the polychoric correlation matrix of the items and using weighted least squares, and the solution is rescaled using oblique factor rotation (*oblimin*). We use the **R** package psych, version 1.8.4 (Revelle, 2018).

**Table A7:** Suggested number of factors to retain – Full set of items for BCS and MCS.

Approach	Rutter (BCS)	SDQ (MCS)
Optimal coordinates	5	3
Acceleration factor	1	1
Parallel analysis	5	5
Kaiser	5	5
VSS Compl. 1	1	1
VSS Compl. 2	2	2
Velicer MAP	2	3

*Notes*: The table compares the optimal number of factors suggested by different approaches: scree test based approaches (optimal coordinates, acceleration factor – Raîche et al., 2013), parallel analysis (Horn, 1965), Kaiser's eigenvalue rule (Kaiser, 1960), Very Simple Structure (VSS, Revelle and Rocklin, 1979), Velicer Minimum Average Partial test (MAP, Velicer, 1976).

**Table A8:** Loadings from exploratory factor analysis – full set of BCS items.

Item	Title	Factor 1	Factor 2	Factor 3	Factor 4	Factor 5
1	Restless*	**0.545**	−0.001	0.061	−0.022	0.042
2	Squirmy/Fidgety*	**0.465**	0.079	0.081	0.035	0.052
3	Destroys Belongings	**0.721**	−0.124	0.035	0.014	−0.006
4	Fights**	**0.684**	−0.046	−0.056	−0.036	−0.029
5	Not Liked	**0.418**	0.219	0.038	−0.046	−0.013
6	Worried*	−0.058	**0.734**	0.018	0.016	0.037
7	Solitary*	0.115	**0.347**	0.001	−0.069	−0.023
8	Irritable	**0.513**	0.269	0.049	−0.066	−0.039
9	Unhappy*	0.295	**0.437**	0.072	0.022	0.028
10	Steals	**0.625**	−0.108	−0.044	0.031	−0.043
11	Twitches	0.215	0.2	0.038	0.027	0.008
12	Sucks thumbs	0.005	0.008	0.002	**0.974**	−0.003
13	Bites Nails	0.002	−0.001	−0.002	−0.003	**0.965**
14	Disobedient*	**0.714**	0.004	−0.001	0.024	0.034
15	Distracted*	**0.547**	0.071	0.042	0.047	0.052
16	Fearful*	−0.042	**0.591**	−0.045	0.057	−0.026
17	Fussy	0.037	**0.462**	0.009	−0.042	0.004
18	Lies	**0.616**	−0.022	−0.013	0.011	0.038
19	Bullies**	**0.659**	0.029	−0.099	−0.005	0.02
A + B	Headaches, stomachaches**	−0.007	−0.011	**0.991**	0.003	0
D	Tantrums**	**0.524**	0.139	0.139	−0.036	−0.017

*Notes*: The table displays the factor loadings obtained from exploratory factor analysis (EFA) on the full set of Rutter items from the BCS. Items denoted by * are used in the 11-item scale. The EFA is performed decomposing the polychoric correlation matrix of the items and using weighted least squares, and the solution is rescaled using oblique factor rotation (*oblimin*). We use the **R** package psych, version 1.8.4 (Revelle, 2018).

**Table A9:** Loadings from exploratory factor analysis – full set of MCS items.

Item	Title	Factor 1	Factor 2	Factor 3	Factor 4	Factor 5
1	Considerate	**0.643**	−0.03	0.028	−0.192	0.055
2	Restless*	0.084	**0.673**	−0.01	0.242	0.1
3	Headaches/stomachaches*	0.09	−0.031	**0.414**	0.185	0.051
4	Shares	**0.571**	−0.055	0.016	−0.112	−0.048
5	Tantrums*	−0.099	0.176	0.244	**0.424**	−0.098
6	Solitary*	−0.025	0.039	**0.328**	−0.094	**0.37**
7	Obedient*	**0.424**	−0.242	0.045	**−0.303**	0.061
8	Worried*	−0.031	−0.044	**0.745**	0.01	0.031
9	Helpful	**0.728**	0.038	−0.042	−0.006	0.025
10	Squirmy/Fidgety*	0.107	**0.615**	0.111	0.18	0.04
11	Good friend	**0.415**	−0.053	−0.002	0.095	**−0.43**
12	Fights/Bullies*	−0.194	0.13	0.082	**0.492**	0.167
13	Unhappy*	−0.038	0.003	**0.591**	0.189	0.113
14	Liked	**0.539**	−0.036	−0.025	−0.048	**−0.404**
15	Distracted*	0.072	**0.838**	0.062	−0.035	0.031
16	Clingy	−0.048	0.032	**0.615**	−0.101	−0.083
17	Kind	**0.658**	0.037	−0.026	−0.075	−0.103
18	Lies	−0.059	0.083	0.118	**0.562**	−0.073
19	Bullied	0.047	0.081	0.266	0.142	**0.349**
20	Volunteers	**0.684**	−0.045	−0.1	0.144	0.084
21	Thinks out	**0.332**	**−0.473**	0.036	−0.027	0.177
22	Steals	−0.111	0.026	−0.004	**0.441**	0.082
23	Adults	0.074	0.128	0.157	0.036	**0.527**
24	Fearful*	0.009	0.053	**0.718**	−0.037	−0.031
25	Sees through	0.236	**−0.714**	0.053	0.131	0.027

*Notes*: The table displays the factor loadings obtained from exploratory factor analysis (EFA) on the full set of SDQ items from the MCS. Items denoted by * are used in the 11-item scale. The EFA is performed decomposing the polychoric correlation matrix of the items and using weighted least squares, and the solution is rescaled using oblique factor rotation (*oblimin*). We use the **R** package psych, version 1.8.4 (Revelle, 2018).

**Table A10:** Measurement invariance fit comparison – single factor.

		Absolute fit						Relative fit					
Model	Num. par.	*χ*^2^	RMSE	SRMR	MFI	CFI	G-hat	*χ*^2^*p*	RMSE	SRMR	MFI	CFI	G-hat
	(1)	(2)	(3)	(4)	(5)	(6)	(7)	(8)	(9)	(10)	(11)	(12)	(13)
Configural	120	4595.9	0.0815	0.0969	0.8640	0.8325	0.9495						
Threshold + Loading Inv	87	5204.7	0.0795	0.1003	0.8477	0.8427	0.9433	0.0000	−0.0020	0.0035	−0.0163	0.0102	−0.0062
Thr. + Load. + Intercept Inv	60	10,192.8	0.1057	0.1049	0.7194	0.6811	0.8931	0.0000	0.0241	0.0080	−0.1446	−0.1514	−0.0565

*Notes*: The table presents fit indices for models of different invariance levels, following Wu and Estabrook (2016) Col. (1) displays the number of estimated parameters for each model. Col. (2) and (8) present the value of the *χ*^2^ statistic and the pvalue of the test of equality with respect to the configural model. Col. (3)–(7) and (9)–(13) present alternative fit indices (AFIs), in absolute values and differences from the configural model respectively. RMSE = Root mean squared error of approximation; SRMR = standardised root mean residual; MFI = McDonald non-centrality index; CFI = comparative fit index; G-hat = gamma-hat.

**Table A11:** Measurement invariance fit comparison – two factors.

		Absolute fit						Relative fit					
Model	Num. par.	*χ*^2^	RMSE	SRMR	MFI	CFI	G-hat	*χ*^2^*p*	RMSE	SRMR	MFI	CFI	G-hat
	(1)	(2)	(3)	(4)	(5)	(6)	(7)	(8)	(9)	(10)	(11)	(12)	(13)
A: Entire sample													
Configural	124	1940.5	0.0522	0.0660	0.9432	0.9333	0.9792						
Threshold + Loading Inv	97	2272.5	0.0525	0.0691	0.9337	0.9282	0.9757	0.0000	0.0003	0.0031	−0.0095	−0.0051	−0.0035
Thr. + Load. + Intercept Inv	70	7293.6	0.0910	0.0754	0.7915	0.7622	0.9217	0.0000	0.0388	0.0094	−0.1517	−0.1711	−0.0575
B: 59–61 months sample													
Configural	124	1596.1	0.0535	0.0674	0.9404	0.9255	0.9781						
Threshold + Loading Inv	97	1784.1	0.0524	0.0696	0.9339	0.9249	0.9757	0.0000	−0.0010	0.0022	−0.0065	−0.0006	−0.0024
Thr. + Load. + Intercept Inv	70	4638	0.0821	0.0748	0.8266	0.7985	0.9353	0.0000	0.0286	0.0074	−0.1138	−0.1271	−0.0429
C: Excluding items 5 and 11													
Configural	108	1258.1	0.0542	0.0634	0.9625	0.9467	0.9833						
Threshold + Loading Inv	81	1685	0.0560	0.0719	0.9499	0.9418	0.9777	0.0000	0.0018	0.0084	−0.0126	−0.0049	−0.0056
Thr. + Load. + Intercept Inv	66	4472.8	0.0886	0.0754	0.8666	0.8170	0.9402	0.0000	0.0344	0.0120	−0.0959	−0.1297	−0.0431

*Notes*: The table presents fit indices for models of different invariance levels, following Wu and Estabrook (2016) Col. (1) displays the number of estimated parameters for each model. Col. (2) and (8) present the value of the *χ*^2^ statistic and the *p*-value of the test of equality with respect to the configural model. Col. (3)–(7) and (9)–(13) present alternative fit indices (AFIs), in absolute values and differences from the configural model respectively. RMSE = Root mean squared error of approximation; SRMR = standardised root mean residual; MFI = McDonald non-centrality index; CFI = comparative fit index; G-hat = gamma-hat. Panel A shows results for the whole sample of children in the BCS and MCS cohorts. Panel B is restricted to a subsample of children in the age range of maximum overlap between the two cohorts (59–61 months). Panel C shows results from a model excluding items 5 and 11 of the 11-item subscale.

**Table A12:** Measurement invariance fit comparison – separate genders.

		Absolute fit						Relative fit					
Model	Num. par.	*χ*^2^	RMSE	SRMR	MFI	CFI	G-hat	*χ*^2^*p*	RMSE	SRMR	MFI	CFI	G-hat
	(1)	(2)	(3)	(4)	(5)	(6)	(7)	(8)	(9)	(10)	(11)	(12)	(13)
A: Males only													
Configural	62	988	0.0520	0.0651	0.9435	0.9376	0.9793						
Threshold + Loading Inv	53	1138	0.0532	0.0676	0.9349	0.9305	0.9761	0.0000	0.0012	0.0025	−0.0085	−0.0072	−0.0032
Thr. + Load. + Intercept Inv	44	3722.8	0.0948	0.0729	0.7917	0.7634	0.9217	0.0000	0.0427	0.0078	−0.1517	−0.1743	−0.0575
B: Females only													
Configural	62	952.6	0.0523	0.0669	0.9429	0.9274	0.9791						
Threshold + Loading Inv	53	1122.4	0.0542	0.0704	0.9326	0.9173	0.9753	0.0000	0.0019	0.0034	−0.0102	−0.0101	−0.0038
Thr. + Load. + Intercept Inv	44	3392.2	0.0927	0.0769	0.7999	0.7415	0.9249	0.0000	0.0404	0.0100	−0.1429	−0.1859	−0.0541

*Notes*: The table presents fit indices for models of different invariance levels, following Wu and Estabrook (2016) Col. (1) displays the number of estimated parameters for each model. Col. (2) and (8) present the value of the *χ*^2^ statistic and the *p*-value of the test of equality with respect to the configural model. Col. (3)–(7) and (9)–(13) present alternative fit indices (AFIs), in absolute values and differences from the configural model respectively. RMSE = Root mean squared error of approximation; SRMR = standardised root mean residual; MFI = McDonald non-centrality index; CFI = comparative fit index; G-hat = gamma-hat. Panel A shows results for the whole sample of male children in the BCS and MCS cohorts. Panel B shows the same for female children.

**Table A13:** Parameter estimates from factor model with threshold and loading invariance.

Panel A: Measurement parameters										
		Loadings	Thresholds		Intercepts (BCS M = 0)			Variances (BCS M = 1)		
		*λ*	*τ*_1_	*τ*_2_	*ν*			diag(*Ψ*)		
Item	Factor	All	All	All	BCS F	MCS M	MCS F	BCS F	MCS M	MCS F
1	EXT	1.218	−0.716	0.894	0.329	1.123	1.522	1.141	0.747	1.031
2	EXT	1.005	−1.642	−0.211	0.014	0.206	0.421	0.873	0.774	0.808
3	EXT	0.635	−1.727	−0.155	0.485	1.350	1.957	1.115	1.009	1.206
4	EXT	0.783	−1.842	−0.376	0.181	−0.580	−0.196	0.880	0.761	0.793
5	EXT	0.669	−0.788		0.261	−0.744	−0.604	1.000	1.000	1.000
6	EXT	0.685	0.747		0.303	0.836	1.104	1.000	1.000	1.000
7	INT	0.763	−1.995	−0.503	−0.101	1.108	0.990	0.858	1.195	0.934
8	INT	0.501	−1.716	−0.362	0.012	−0.129	−0.254	0.978	1.170	0.914
9	INT	0.391	−1.398	−0.107	0.060	0.454	0.586	0.941	0.906	1.043
10	INT	1.128	−3.030	−1.251	−0.206	1.056	1.076	1.030	0.718	1.012
11	INT	0.423	−1.249		−0.094	−0.011	−0.317	1.000	1.000	1.000


Panel B: Latent variable parameters								
	Mean		Covariance				Correlation	
	*κ*		*Φ*					
	BCS	MCS	BCS		MCS		BCS	MCS
Males								
*θ*^*EXT*^	0.000	0.000	1.000		1.325			
*θ*^*INT*^	0.000	0.000	0.421	1.000	0.833	1.817	0.421	0.537
Females								
*θ*^*EXT*^	0.000	0.000	0.985		1.120			
*θ*^*INT*^	0.000	0.000	0.478	1.012	0.557	1.388	0.479	0.447

*Notes*: The table presents estimates for the factor model with loadings *λ* and thresholds *τ* restricted to be equal across cohorts. Panel A shows estimates of the measurement parameters. Loadings and thresholds are the same across all cohorts. Intercepts are restricted to zero in the reference group, i.e. males in BCS (not shown). Variances of the error terms are restricted to one in the reference group, i.e. males in BCS (not shown), and for the items that only have two categories (5, 6, 11). Panel B shows estimates of the latent variable parameters. Means are restricted to zero in all cohort-gender groups, while variances are restricted to one only in the reference group, i.e. males in BCS.

**Table A14:** RIF decomposition results - externalising skills, male sample.

Inequality measures	90–10	75–25	50–10	90–50	Variance
Total difference	0.367***	0.327***	0.244***	0.123**	0.254***
Composition effect	0.170*	0.123**	0.113*	0.056	0.073**
Coefficient effect	0.098	0.185	0.159	−0.061	0.186***
*Composition effects:*					
Mother post-compulsory education (5)	−0.003	0.001	−0.019*	0.015	0.001
Mother employed (5)	0.004	−0.006	0.001	0.002	−0.001
Father blue-collar occupation (5)	−0.019	−0.011	0.000	−0.019	−0.009
No father fig. (5)	0.033	0.059*	−0.014	0.047	0.015
Mother's age	0.081**	0.058**	0.056*	0.025	0.019
Mother's height	−0.013	0.001	−0.007	−0.006	−0.004
Mother unmarried	0.068	0.009	0.063	0.006	0.045*
Non-white ethnicity	0.010	−0.001	0.013	−0.003	−0.002
No. other children in household (5)	−0.002	−0.002	−0.002	−0.001	−0.001
Child firstborn	−0.001	0.001	0.000	−0.001	0.000
No. previous stillbirths	0.004	0.000	0.003	0.001	0.001
Mother smoked in pregnancy	−0.002	0.009	−0.004	0.002	−0.001
Child born preterm	0.014	0.004	0.014	0.000	0.007
Gestational age missing	−0.003	0.005	0.003	−0.006	0.006
Log birth weight	−0.001	−0.004	0.005	−0.006	−0.001
Specification error	0.158	0.168	0.031	0.128	0.059
*Coefficient effects:*					
Mother post-compulsory education (5)	−0.137	0.040	−0.229	0.092	−0.047
Mother employed (5)	−0.046	−0.350**	0.186	−0.232*	−0.112
Father blue-collar occupation (5)	−0.158	−0.119	−0.115	−0.042	−0.079
No father fig. (5)	−0.092	−0.114	−0.099	0.007	−0.048
Mother's age	−0.795	−0.711	−0.684	−0.111	−0.579*
Mother's height	−0.929	−5.180	3.978	−4.906*	−0.530
Mother unmarried	0.140	0.163	0.096	0.044	0.061
Non-white ethnicity	0.052	−0.104	0.101	−0.050	−0.004
No. other children in household (5)	−0.146	0.127	−0.091	−0.055	−0.047
Child firstborn	−0.208*	−0.192	−0.058	−0.150	−0.136**
No. previous stillbirths	0.002	0.001	0.004	−0.002	0.001
Mother smoked in pregnancy	0.051	−0.095	0.005	0.046	0.005
Child born preterm	0.044	0.000	0.002	0.042	0.023
Gestational age missing	0.001	−0.002	0.002	−0.002	0.000
Log birth weight	0.282	0.382	−0.297	0.579	−0.137
Constant	2.038	6.338*	−2.643	4.680	1.816
Reweighting error	−0.059	−0.149	−0.060	0.000	−0.065

*Notes*: The table shows the detailed results of the RIF Decomposition (with reweighting) as in Firpo et al. (2018). Bootstrapped standard errors over the entire procedure (500 replications) are used to compute the *p*-values. ^∗∗∗^p≤0.01, ^∗∗^p≤0.05, ^∗^p≤0.1.

**Table A15:** RIF decomposition results - internalising skills, male sample.

Inequality measures	90–10	75–25	50–10	90–50	Variance
Total difference	0.578***	0.217***	0.391***	0.187***	0.339***
Composition effect	−0.002	−0.083*	0.071	−0.073*	−0.015
Coefficient effect	0.749***	0.306***	0.369***	0.381***	0.365***
*Composition effects*					
Mother post-compulsory education (5)	−0.008	0.002	−0.002	−0.006	−0.002
Mother employed (5)	−0.008	−0.001	−0.003	−0.005	−0.004
Father blue-collar occupation (5)	−0.012	−0.009	−0.008	−0.004	−0.006
No father fig. (5)	0.024	0.020	0.002	0.022	0.014
Mother's age	−0.013	−0.011	−0.005	−0.008	−0.010
Mother's height	−0.006	−0.001	0.000	−0.006	−0.003
Mother unmarried	0.005	−0.074**	0.071	−0.066**	−0.010
Non-white ethnicity	0.016	0.013	0.029	−0.013	0.006
No. other children in household (5)	−0.009	−0.003	−0.002	−0.007	−0.003
Child firstborn	0.009	0.003	0.001	0.008	0.002
No. previous stillbirths	−0.002	−0.002	0.000	−0.002	0.000
Mother smoked in pregnancy	−0.009	−0.009	−0.009	0.000	−0.002
Child born preterm	−0.019	−0.024	−0.030	0.011	−0.007
Gestational age missing	0.028***	0.016**	0.025***	0.003	0.012***
Log birth weight	0.003	−0.003	0.004	−0.001	−0.001
Specification Error	−0.084	−0.015	0.044	−0.128	−0.026
*Coefficient effects*					
Mother post-compulsory education (5)	0.111	0.193	0.220	−0.109	0.041
Mother employed (5)	−0.327**	−0.168	−0.357***	0.030	−0.187***
Father blue-collar occupation (5)	−0.059	−0.120*	−0.096	0.036	−0.043
No father fig. (5)	0.063	0.019	0.014	0.048	0.010
Mother's age	−0.987	−0.086	−1.094**	0.107	−0.281
Mother's height	−0.190	−1.952	−1.826	1.636	−0.019
Mother unmarried	0.096	0.155*	0.005	0.091	0.044
Non-white ethnicity	0.002	0.045	−0.009	0.011	0.023
No. other children in household (5)	0.125	0.042	0.259	−0.134*	−0.016
Child firstborn	−0.051	−0.031	0.003	−0.054	0.000
No. previous stillbirths	0.004	−0.002	0.006	−0.002	0.003
Mother smoked in pregnancy	−0.081	−0.039	−0.055	−0.025	−0.022
Child born preterm	−0.014	0.009	0.010	−0.024	0.025*
Gestational age missing	0.003	−0.003	0.002	0.001	0.002
Log birth weight	−1.068	0.413	−0.213	−0.855	−0.217
Constant	3.124	1.831	3.501	−0.377	1.003
Reweighting error	−0.085	0.010	−0.093	0.008	0.015

*Notes*: The table shows the detailed results of the RIF Decomposition (with reweighting) as in Firpo et al. (2018). Bootstrapped standard errors over the entire procedure (500 replications) are used to compute the *p*-values. ^∗∗∗^p≤0.01, ^∗∗^p≤0.05, ^∗^p≤0.1.

**Table A16:** RIF decomposition results - externalising and internalising skills, female sample.

	Externalising		Internalising	
Inequality measures	50–10	Variance	50–10	Variance
Total difference	0.132***	0.056***	0.109**	0.062***
Composition effect	−0.022	0.010	0.059	0.014
Coefficient effect	0.137	0.085**	−0.010	0.054
*Composition effects*				
Mother post-compulsory education (5)	0.006	−0.005	0.005	−0.001
Mother employed (5)	−0.013	−0.006	−0.014	−0.006
Father blue-collar occupation (5)	−0.006	−0.002	−0.003	−0.003
No father fig. (5)	0.055	0.061***	0.043	0.031
Mother's age	0.032	0.013	0.041**	0.011
Mother's height	−0.006	−0.005	0.002	−0.003
Mother unmarried	−0.095**	−0.039*	−0.008	−0.002
Non-white ethnicity	0.034**	0.014*	−0.001	−0.002
No. other children in household (5)	−0.004	−0.003	0.000	−0.002
Child firstborn	0.000	0.000	−0.001	0.004
No. previous stillbirths	0.000	0.000	0.002	0.001
Mother smoked in pregnancy	−0.011	−0.010**	0.001	−0.004
Child born preterm	−0.005	−0.003	0.006	0.000
Gestational age missing	−0.012	−0.005	−0.012	−0.008
Log birth weight	0.004	−0.001	−0.002	−0.002
Specification Error	0.000	0.023	0.192	0.050
*Coefficient effects*				
Mother post-compulsory education (5)	−0.181	0.013	−0.286	0.050
Mother employed (5)	−0.030	−0.108**	−0.200	−0.156**
Father blue-collar occupation (5)	−0.113	−0.033	−0.094	−0.027
No father fig. (5)	−0.038	−0.047**	−0.065	−0.014
Mother's age	−0.479	−0.389*	−0.599	−0.064
Mother's height	−0.584	1.231	5.447	4.406**
Mother unmarried	0.076	0.069**	−0.048	−0.010
Non-white ethnicity	0.004	−0.007	−0.010	0.008
No. other children in household (5)	0.112	−0.054	0.266	0.086
Child firstborn	−0.138	−0.050	0.170	0.052
No. previous stillbirths	0.004	0.003	0.013*	0.004*
Mother smoked in pregnancy	0.029	−0.013	0.036	0.027
Child born preterm	0.034	−0.013	−0.082	−0.031**
Gestational age missing	0.001	0.001	0.003	0.002
Log birth weight	−0.484	−0.388	−0.880	−0.311
Constant	1.925	−0.132	−3.680	−3.968*
Reweighting error	0.017	−0.062**	−0.131	−0.057

*Notes*: The table shows the detailed results of the RIF Decomposition (with reweighting) as in Firpo et al. (2018). Bootstrapped standard errors over the entire procedure (500 replications) are used to compute the *p*-values. ^∗∗∗^ p≤0.01, ^∗∗^ p≤0.05, ^∗^ p≤0.1.

**Table A17:** Predictors of adolescent outcomes, BCS – factor scores vs. sum scores.

	Males				Females			
	Mean	Coefficients			Mean	Coefficients		
	(1)	(2)	(3)	(4)	(5)	(6)	(7)	(8)
*Tried smoking (BCS - 16)*	.524				.586			
Externalising skills (5)		−.073^∗∗∗^	−.081^∗∗∗^			−.068^∗∗∗^	−.077^∗∗∗^	
		(.024)	(.025)			(.021)	(.022)	
Internalising skills (5)		.055^∗∗^	.060^∗∗^			.039	.045^∗^	
		(.028)	(.029)			(.025)	(.026)	
Externalising (sum score)				−.074^∗∗∗^				−.067^∗∗∗^
				(.024)				(.021)
Internalising (sum score)				.061^∗^				.036
				(.033)				(.026)
Cognitive skills (5)			.010	.010			.012	.012
			(.019)	(.020)			(.017)	(.017)
Adj. R^2^		0.032	0.032	0.033		0.048	0.046	0.045
Observations		1197	1123	1123		1693	1581	1581
*BMI (BCS - 16)*	20.9				21.2			
Externalising skills (5)		−.178	−.227^∗^			−.225^∗^	−.222^∗^	
		(.118)	(.122)			(.122)	(.128)	
Internalising skills (5)		.036	.062			.280^∗∗^	.234	
		(.140)	(.146)			(.141)	(.145)	
Externalising (sum score)				−.246^∗∗^				−.190
				(.125)				(.122)
Internalising (sum score)				−.003				.233
				(.170)				(.156)
Cognitive skills (5)			.021	.023			−.093	−.092
			(.101)	(.102)			(.101)	(.101)
Adj. R^2^		0.017	0.018	0.018		0.023	0.023	0.023
Observations		1640	1531	1531		1873	1757	1757

*Notes*: The table shows coefficients from linear regressions of cohort members' adolescent outcomes on their externalising and internalising socio-emotional skills at five years of age. Col. (1) shows the mean of the outcome for males. Col. (2) regresses the outcome on the scores obtained from the factor model in Section 4. Col. (3) additionally controls for cognitive ability at age five. This is a simple factor score obtained by aggregating the available cognitive measures. All standard errors in parentheses are obtained using 1000 bootstrap repetitions, taking into account the factor estimation stage that precedes the regression. Col. (4) replaces the factor scores used in col. (3) with simpler sum scores – see Fig. A1. Col. (5) to (8) repeat for female cohort members. All estimates additionally control for region of birth, maternal education (5), maternal employment (5), father occupation (5), maternal background (age, height, nonwhite ethnicity, number of children in the household), pregnancy (firstborn child, number of previous stillbirths, mother smoked in pregnancy, preterm birth, (log) birth weight). See Table A1 for a description of the variables used. ^∗∗∗^ p≤0.01, ^∗∗^ p≤0.05, ^∗^ p≤0.1.

**Table A18:** Predictors of adolescent outcomes, maximum likelihood estimates.

	Males	Females
*Tried smoking (BCS - 16)*		
Externalising skills (5)	−.128^∗∗∗^	−.157^∗∗∗^
	(.042)	(.039)
Internalising skills (5)	.084^∗^	.080^∗∗^
	(.046)	(.035)
Observations	1575	2133
*BMI (BCS - 16)*		
Externalising skills (5)	−.211^∗∗^	−.158
	(.093)	(.103)
Internalising skills (5)	.071	.127^∗^
	(.104)	(.167)
Observations	2011	2295
*Tried smoking (MCS - 14)*		
Externalising skills (5)	−.063^∗∗^	−.049
	(.028)	(.034)
Internalising skills (5)	.010	.058
	(.043)	(.056)
Observations	1997	2024
*BMI (MCS - 14)*		
Externalising skills (5)	−.269^∗∗^	−.439^∗∗∗^
	(.133)	(.153)
Internalising skills (5)	.091	.510^∗∗^
	(.201)	(.246)
Observations	2005	1936

*Notes*: The table shows coefficients from the joint estimation of the (partially invariant) measurement system for externalising and internalising socio-emotional skills at five years of age and of the cohort members' adolescent outcomes. Estimation is by maximum likelihood, see Table 6 for the corresponding results obtained via the two-step process. All estimates control for region of birth, maternal education (5), maternal employment (5), father occupation (5), maternal background (age, height, nonwhite ethnicity, number of children in the household), pregnancy (firstborn child, number of previous stillbirths, mother smoked in pregnancy, preterm birth, (log) birth weight). See Table A1 for a description of the variables used. ^∗∗∗^ p≤0.01, ^∗∗^ p≤0.05, ^∗^ p≤0.1.

**Table A19:** Predictors of adult behaviours, BCS.

	Males				Females			
	Mean	Coefficients			Mean	Coefficients		
	(1)	(2)	(3)	(4)	(5)	(6)	(7)	(8)
*Daily smoker (42)*	.180				.147			
Externalising skills (5)		−.062^∗∗∗^	−.059^∗∗∗^			−.050^∗∗∗^	−.049^∗∗∗^	
		(.017)	(.017)			(.015)	(.016)	
Internalising skills (5)		.027	.025			.043^∗∗∗^	.048^∗∗∗^	
		(.020)	(.020)			(.017)	(.017)	
Externalising (sum score)				−.059^∗∗∗^				−.040^∗∗∗^
				(.018)				(.015)
Internalising (sum score)				.008				.049^∗∗∗^
				(.024)				(.019)
Cognitive skills (5)			−.022	−.022			−.032^∗∗∗^	−.032^∗∗∗^
			(.015)	(.014)			(.012)	(.012)
Adj. R^2^		0.044	0.045	0.045		0.037	0.041	0.041
Observations		1294	1216	1216		1678	1572	1572
	
*BMI (42)*	27.5				26.1			
Externalising skills (5)		−.267	−.138			−.385	−.242	
		(.227)	(.234)			(.252)	(.262)	
Internalising skills (5)		.400	.316			.102	−.035	
		(.274)	(.282)			(.292)	(.300)	
Externalising (sum score)				−.060				−.299
				(.234)				(.258)
Internalising (sum score)				.277				−.081
				(.320)				(.328)
Cognitive skills (5)			−.235	−.235			−.729^∗∗∗^	−.728^∗∗∗^
			(.186)	(.192)			(.226)	(.214)
Adj. R^2^		0.028	0.024	0.024		0.034	0.047	0.047
Observations		1149	1078	1078		1399	1317	1317

*Notes*: The table shows coefficients from linear regressions of cohort members' adolescent and adult outcomes on their externalising and internalising socio-emotional skills at five years of age. Col. (1) shows the mean of the outcome for males. Col. (2) regresses the outcome on the scores obtained from the factor model in Section 4. Col. (3) additionally controls for cognitive ability at age five. This is a simple factor score obtained by aggregating the available cognitive measures. Col. (4) uses sum scores (see Fig. A1) instead of factor scores. All standard errors in parentheses are obtained using 1000 bootstrap repetitions, taking into account the factor estimation stage that precedes the regression. Col. (5) to (8) repeat for female cohort members. All estimates additionally control for region of birth, maternal education (5), maternal employment (5), father occupation (5), maternal background (age, height, nonwhite ethnicity, number of children in the household), pregnancy (firstborn child, number of previous stillbirths, mother smoked in pregnancy, preterm birth, (log) birth weight). See Table A1 for a description of the variables used. ^∗∗∗^ p≤0.01, ^∗∗^ p≤0.05, ^∗^ p≤0.1.

**Fig. A2:**
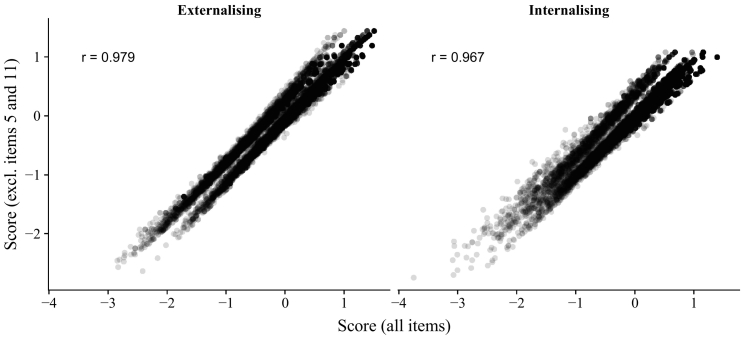
Factor scores excluding items 5 and 11. *Notes*: The figure shows the relationship between factor scores from the full 11-item model (horizontal axis) against factors scores from a model excluding items 5 and 11 (vertical axis). Pearson correlations are reported in the plot area.

**Fig. A3:**
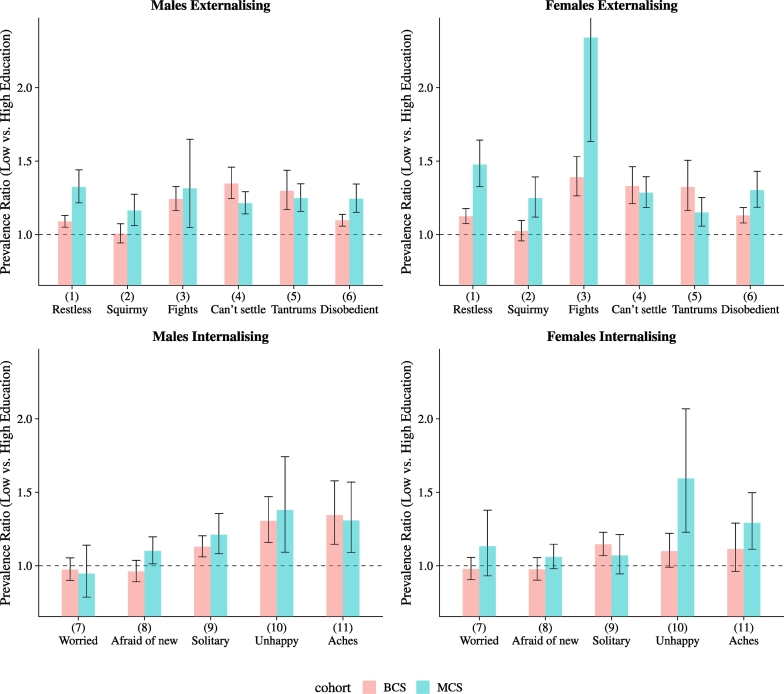
Item-level inequality by mother's education. *Notes*: The graph displays the ratio between the prevalence of each item in our scale in children of educated vs uneducated mothers, by cohort and gender. All items that have three categories in the scale have been dichotomised. For example, if the prevalence of the ‘Restless’ behaviours among children of mothers with compulsory schooling in the BCS cohort is 7.5%, and 5% among mothers with post-compulsory schooling, the ratio will be 1.5. The error bars represent 95% confidence intervals.

**Fig. A4:**
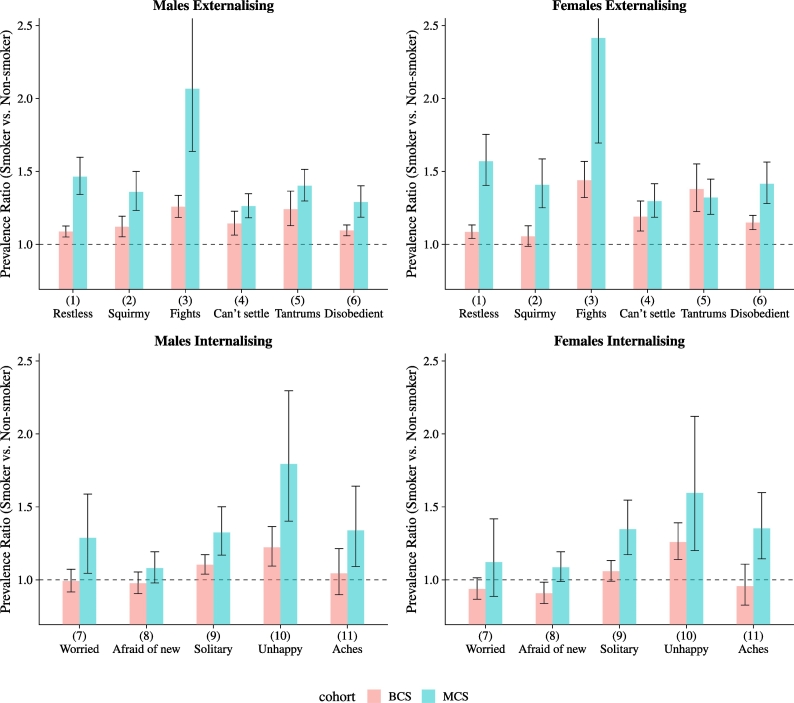
Item-level inequality by mother's pregnancy smoking. *Notes*: The graph displays the ratio between the prevalence of each item in our scale in children of mothers who smoked in pregnancy vs non-smokers, by cohort and gender. All items that have three categories in the scale have been dichotomised. For example, if the prevalence of the ‘Restless’ behaviours among children of smoker mothers in the BCS cohort is 7.5%, and 5% among non-smoker mothers, the ratio will be 1.5. The error bars represent 95% confidence intervals.

**Fig. A5:**
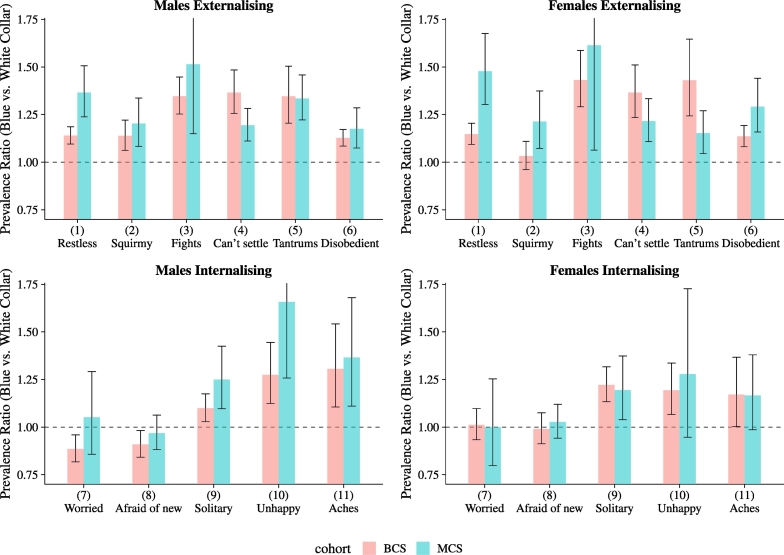
Item-level inequality by father's occupation. *Notes*: The graph displays the ratio between the prevalence of each item in our scale in children of white collar vs blue collar fathers, by cohort and gender. All items that have three categories in the scale have been dichotomised. For example, if the prevalence of the ‘Restless’ behaviours among children of blue collar fathers in the BCS cohort is 7.5%, and 5% among white collar fathers, the ratio will be 1.5. The error bars represent 95% confidence intervals. (For interpretation of the references to colour in this figure legend, the reader is referred to the web version of this article.)

## Appendix A Deriving a common scale of socio-emotional skills

In the BCS, maternal reports on child socio-emotional skills are measured using the Rutter A Scale (Rutter et al., 1970) – see Panel A of Table A2. The Rutter items are rated on three levels: ‘Does not apply’, ‘Somewhat applies’, ‘Certainly applies’. Since they all indicate negative behaviours, we recode all of them in reverse, i.e. ‘Certainly applies’ = 0, ‘Somewhat applies’ = 1, ‘Does not apply’ = 2. We augment the 19-item Rutter Scale with three additional parent-reported questions from the parental questionnaire, items A, B, and D. These are rated on 4 levels: ‘Never in the last 12 months’, ‘less than once a month’, ‘at least once a month’, ‘at least once a week’; we recode them into binary indicators, with ‘Never’ and ‘Less than once a month’ to 1 and zero otherwise. To increase comparability between the two scales, we also merge together two pairs of Rutter items: 4 and 19 (to mirror SDQ item 12 “Often fights with other children or bullies them”), and A and B (to mirror SDQ item 3 “Often complains of head-aches, stomach-ache or sickness”); we assign the lowest category among the two original items to the newly obtained item. We also recode the three-category Rutter items 5 and 14 to binary to mimic the split in the MCS, where they are worded positively.

In the MCS, we use the 25-item Strengths and Difficulties Questionnaire (Goodman, 1997) – see Panel B of Table A2. All items are recorded on a 4-point scale: ‘Not true’, ‘Somewhat true’, ‘Certainly true’, ‘Can't say’. We set the latter option to missing and recode the rest so that a greater value represents a higher level of skills, as for the BCS items, i.e. ‘Certainly true’ = 0, ‘Somewhat true’ = 1, ‘Not true’ = 2 for the negatively-worded items (and the opposite for the positively-worded ones). For comparability with the BCS Rutter scale, we dichotomise items 3 and 5, and dichotomise and invert items 7 and 14.

## Appendix B Robustness of exploratory analysis

In this section, we repeat the exploratory analysis step in Section 4.1 for the full set of Rutter and SDQ items. This is to show that the factor structure emerging from the exploratory analysis of the 11-item subscale is consistent with what would emerge considering the original scales in their entirety. Again, we proceed by first assessing the optimal number of factors, and then examining the loadings obtained from exploratory factor analysis.

Results for the optimal number of factors as indicated by different approaches are in Table A7. Similarly to the 11-item subscale, there is not much agreement between methods. Since the purpose of this section is to assess the robustness of the 11-item subscale, we adopt a conservative approach by estimating EFA models with the largest number of factors suggested, i.e. five. In this way, we allow for richer factor solutions, that have more power to disprove our simpler two-factor solution for the novel subscale.

Table A8 presents factor loadings for the Rutter scale in BCS, with the addition of the “headaches/stomachaches” and “tantrums” items (see Appendix A). The split between externalising and internalising items that we recover in the 11-item scale is almost entirely preserved in the full scale, as seen by items loading on factors 1 and 2. The only exception is the “headaches/stomachaches” item, which seems to load on a separate factor. We then carry out robustness checks for the measurement invariance analysis excluding this item in Appendix C.

The same analysis is repeated for the SDQ scale in MCS in Table A9. An internalising, emotional dimension (factor 3) emerges neatly, and coherently with the analysis on our subscale. The externalising items from our subscales are split across two dimensions in this full-scale EFA: one more related to hyperactivity (factor 2) and one to conduct problems (factor 4). This is consistent with the original structure of the SDQ (Goodman, 1997).

## Appendix C Robustness of item choice

In deriving our novel 11-item scale, we construct two items for the BCS cohort based on questions that are not in the original Rutter scale – namely those concerning “headaches/stomachaches” and “tantrums” (see Appendix A above for details). Concerns might arise that introducing these items might somewhat invalidate our main conclusions, rather than provide additional informational content on children's externalising and internalising behaviours and symptoms.

In fact, exploratory factor analysis (on both the full Rutter and SDQ scales and on the 11-item subscale) shows that these items, numbered 5 and 11, perform poorly and exhibit relatively low factor loadings. As a robustness check, we replicate the main results of the paper by excluding them from the subscale.

Panel C of Table A11 shows that the measurement invariance analysis yields the same qualitative results once these two items are included. Fig. A2 shows a scatter plot of the factor scores obtained from the factor model with and without items 5 and 11. They exhibit very high correlation, thus indicating that our results in Section 6 would not substantially change if we omitted the two items with the least informational content.

## Footnotes

1 As the authors note (footnote 41) “Our measures of non-cognitive skills in the two countries are clearly not equivalent. The Danish measure of non-cognitive skills is more related to an orderliness/effort measure while the US measure is related to behavioural problems”.

2 He uses the following four variables as measures of social skills in the NLSY79: self-reported sociability in 1981 and at age 6 (retrospective), the number of clubs in which the respondent participated in high school and participation in high school sports; and the following two variables in the NLSY97: two questions that capture the extroversion factor from the Big 5 Personality Inventory (since measures comparable to the NLSY79 are not available in the NLSY97).

3 Student's effort, initiative, non-participatory behaviour, and how the student is seen to ‘value’ the class.

4 All data is publicly available at the UK Data Service (Chamberlain, 2013; Butler, 2016a, Butler, 2016b, Butler, 2017; University Of London. Institute Of Education. Centre For Longitudinal Studies, 2016a, University Of London. Institute Of Education. Centre For Longitudinal Studies, 2016b, University Of London. Institute Of Education. Centre For Longitudinal Studies, 2016c, University Of London. Institute Of Education. Centre For Longitudinal Studies, 2017a, University Of London. Institute Of Education. Centre For Longitudinal Studies, 2017b, University Of London. Institute Of Education. Centre For Longitudinal Studies, 2017c).

5 See Table 5.5 in Plewis et al. (2007). This choice is mainly driven by software limitations. The lavaan package in **R** (Rosseel, 2012) is the most suitable tool for our invariance analysis, but it does not allow to use weights when outcomes are categorical, as it is the case for the socio-emotional measurements.

6 Of course, we could have included them in the factor analysis and treated them as missing in the cohort where they were not administered.

7 See for example Fowler and Park (1979); Venables et al. (1983); Tremblay et al. (1987); Berglund (1999); Klein et al. (2009). However, in some cases a three-factor structure was found to better fit the data, with the externalising factor separating into two factors seemingly capturing aggressive and hyperactive behaviours (Behar and Stringfield, 1974; McGee et al., 1985).

8 Factor-analytic methods have long been used in psychology, and in recent years they have become increasingly popular in economics, especially to meaningfully aggregate high-dimensional items measuring different aspects of common underlying dimensions of human development. The EFA is performed decomposing the polychoric correlation matrix of the items and using weighted least squares, and the solution is rescaled using oblique factor rotation (*oblimin*). We use the **R** package psych, version 1.8.4 (Revelle, 2018).

9 We do not perform the EFA with 3 factors for males because this solution is never chosen by any test for the number of factors for the MCS, see Table A4.

10 Internalising and externalising dimensions emerge from the exploratory step on our novel 11-item scale. Appendix B performs the same exploratory steps on the full set of Rutter items in BCS and SDQ items in MCS. It confirms that the items we select for our subscale have a broadly consistent covariance structure even when factor-analysed with the others in their original scales. Appendix C considers the robustness of our results to the exclusion of the items of the scale that perform most poorly.

11 The dedicated factor structure in the two-factor case corresponds to a sparse loading matrix, i.e.: $\Lambda_{c}≔\left[\begin{array}{cc} \lambda_{1c},\ldots ,\lambda_{6c} & 0\\ 0 & \lambda_{7c},\ldots ,\lambda_{11c} \end{array}\right].$

12 The model implies the following expression for the mean and covariance structure of the latent propensities: *μ*_*c*_ = *ν*_*c*_ + *Λ*_*c*_*κ*_*c*_ and *Σ*_*c*_ = *Λ*_*c*_*Φ*_*c*_*Λ*_*c*_′ + *Ψ*_*c*_. As per the traditional factor analysis approach, we impose a normal distribution on the latent skills and error terms.

(4.3) $$\theta_{\mathrm{jc}}\sim N\left(\kappa_{c},\Phi_{c}\right)\;\text{and}\;u_{\mathrm{jc}}\sim N\left(0,\Psi_{c}\right).$$

Recent work has also used mixtures of normals for the latent factors distribution, e.g. Conti et al. (2010).

13 We recognise that simultaneous invariance of *all* items is not the minimum requirement for comparability. In theory, the availability of just one invariant item (known as ‘anchor’) would suffice to fix the scale and location of the system. However, partial invariance approaches are hard to implement in practice. Its validity hinges on selecting one (or more) truly invariant anchor, which is challenging on an a priori basis. The full procedure, restricting all parameters of a certain type across groups, does not identify which items are at the source of the invariance. Algorithms have been proposed to deal with this issue (Yoon and Millsap, 2007; Cheung and Lau, 2012), however there are still doubts on their robustness and their applicability to the categorical case (Vandenberg and Morelli, 2016).

14 All estimates are computed using the lavaan package (version 0.6–2) in **R** (Rosseel, 2012).

15 It is worth noting that threshold and loading invariance only can be established also in the 1-factor case, i.e. intercept invariance is never achieved.

16 We do not present fit results for the threshold-only invariance model, as it is statistically equivalent to the configural model and thus its fit is mathematically the same – see Table 3 in Wu and Estabrook, 2016. The ages at which socio-emotional skills are observed varies slightly between BCS and MCS, due to different sampling and fieldwork schedules. In the MCS cohort, the age distribution has significantly higher variance. In Panel B of Table A11, we restrict the sample to 59 to 61 months, where the overlap between BCS and MCS is maximised. In Panel C, we repeat the analysis with the full sample, but excluding the poorest-performing items (5 and 11) – see Appendix C for details. In Panels A and B of Table A12, we restrict to male and female children respectively. In all these cases, invariance of thresholds and loadings is confirmed, but invariance of intercepts is rejected. We can thus rule out that the lack of intercept invariance comes from differences in ages or invariance across child gender.

17 Calibrating the Rutter and SDQ using a contemporary sample of children cannot rule out this issue. For example, Collishaw et al. (2004) administered both Rutter and SDQ items to parents of a small sample of adolescents in London. They use the mapping between the two questionnaires to impute Rutter scores for mothers who answered the SDQ. This can correct for contemporaneous reporting differences between questionnaires, but cannot tackle reporting differences between samples collected at different times in history.

18 We use an empirical Bayes modal (EBM) approach to estimate the scores. The parameters are estimated using three sources of information. The first is the distribution of the latent variables *θ*, treated as random parameters with a prior *h*(*θ*, *Ω*), conditional on the parameters *Ω*. This prior is assumed to be multivariate normal. The second is the observed data *X*, and the third is the estimated parameters $\hat{\Omega }$. Data and prior are combined into the posterior distribution $w(\theta |X,\hat{\Omega })$. For further details, see Chapter 7 in Skrondal and Rabe-Hesketh (2004).

19 Figs. 8, 9, and 10 show inequality in the scale items underlying the factor scores used in this section. The increase in inequality across cohorts is still present, but less marked when looking at these single items. This shows the importance of the factor analysis step in aggregating items, explicitly modelling the measurement error, and testing and accounting for (loadings and thresholds) invariance across the two cohorts.

20 We also estimated Tobit models to account for the right truncation of the distribution of skills – see Fig. 1. Tobit estimates are extremely similar to the linear estimates in Table 5, and are available from the authors upon request.

21 It is important to underscore that there has been a significant rise is cohabitation between 1975 and 2006. It is likely that unmarried mothers in the two cohorts have very different characteristics. The choice of this indicator is due to the absence of information on cohabitation in the birth survey for the BCS cohort.

22 See also Fortin et al. (2011) for a recent survey of decomposition methods in economics.

23 We use a logit model to construct the weights and the post-double selection lasso (Belloni et al., 2014) to select the covariates, among the set of the baseline variables in Table 1 and their pairwise interactions.

24 The coefficient effects are also sizeable, but imprecisely estimated, with the exception of the variance component.

25 In Table A17, Table A19, we show that the conclusions in this section are not sensitive to the factor scoring methodology used. ‘Raw’ scores, obtained by a simple unweighted average of the item categories in the 11-item subscale have basically equal predictive power to factor scores.

26 Unfortunately the strength of the association cannot be directly compared, since the outcomes are measured at different ages: 16 and 14 years for BCS and MCS, respectively.

27 Note that the magnitudes are not exactly comparable because in one-step ML estimation the residual variances of the non-binary measurements also need to be fixed for identification. The remaining parameter estimates are also very similar to those in Table 5 and available from the authors upon request.

28 We do not observe significant associations between early socio-emotional skills and other risky behaviours like drug-taking and alcohol consumption. One possible reason might be the relatively young age at which these skills are measured. Results are available upon request.

29 They use the Rutter scale and the Bristol Social Adjustment Guide.
